# Hypercoagulability Is a Stronger Risk Factor for Ischaemic Stroke than for Myocardial Infarction: A Systematic Review

**DOI:** 10.1371/journal.pone.0133523

**Published:** 2015-08-07

**Authors:** Alberto Maino, Frits R. Rosendaal, Ale Algra, Flora Peyvandi, Bob Siegerink

**Affiliations:** 1 Department of Clinical Epidemiology, Leiden University Medical Center, Leiden, The Netherlands; 2 Angelo Bianchi Bonomi Hemophilia and Thrombosis Center, Fondazione IRCCS Ca’ Granda Ospedale Maggiore Policlinico, Department of Pathophysiology and Transplantation, Università degli Studi di Milano, Milan, Italy; 3 Einthoven Laboratory for Experimental Vascular Medicine, Leiden University Medical Center, Leiden, The Netherlands; 4 Department of Thrombosis and Hemostasis, Leiden University Medical Center, Leiden, The Netherlands; 5 Brain Center Rudolph Magnus, Department of Neurology and Neurosurgery, University Medical Center Utrecht, Utrecht, The Netherlands; 6 Julius Center for Health Sciences and Primary Care, University Medical Center Utrecht, Utrecht, The Netherlands; 7 Center for Stroke Research Berlin, Charité Universitätsmedizin Berlin, Berlin, Germany; Maastricht University Medical Center, NETHERLANDS

## Abstract

**Background and Purpose:**

Hypercoagulability increases the risk of arterial thrombosis; however, this effect may differ between various manifestations of arterial disease.

**Methods:**

In this study, we compared the effect of coagulation factors as measures of hypercoagulability on the risk of ischaemic stroke (IS) and myocardial infarction (MI) by performing a systematic review of the literature. The effect of a risk factor on IS (relative risk for IS, RR_IS_) was compared with the effect on MI (RR_MI_) by calculating their ratio (RRR = RR_IS_/RR_MI_). A relevant differential effect was considered when RRR was >1+ its own standard error (SE) or <1−SE.

**Results:**

We identified 70 publications, describing results from 31 study populations, accounting for 351 markers of hypercoagulability. The majority (203/351, 58%) had an RRR greater than 1. A larger effect on IS risk than MI risk (RRE>1+1SE) was found in 49/343 (14%) markers. Of these, 18/49 (37%) had an RRR greater than 1+2SE. On the opposite side, a larger effect on MI risk (RRR<1-1SE) was found in only 17/343 (5%) markers.

**Conclusions:**

These results suggest that hypercoagulability has a more pronounced effect on the risk of IS than that of MI.

## Introduction

Myocardial infarction (MI) and ischaemic stroke (IS), the main manifestations of arterial thrombosis, are the most common causes of morbidity and mortality globally [1, 2]. Many risk factors are shared by both diseases because the pathophysiologic mechanism is similar: the formation of a thrombus in the arteries supplying oxygen to either the heart or the brain [3]. Platelets play a pivotal role in the formation and propagation of the thrombus, and therefore are the primary targets of antithrombotic therapy in arterial disease [4]. However, arterial thrombus formation is also determined by the activation of the coagulation cascade [5–7]. These two mechanisms work intertwined and independently: thrombin transforms fibrinogen into fibrin, but also activates platelets, both important factors in thrombus growth and stability. As a consequence, drugs that target thrombin generation (vitamin K antagonists and FXa inhibitors), thrombin’s catalytic function (direct thrombin inhibitors) or thrombin’s activation of platelets (PAR1 antagonists) all inhibit arterial thrombosis [8].

Hypercoagulability, a condition in which the haemostatic balance is tilted towards thrombus formation, increases the risk of arterial thrombosis [9]. An increased risk of both MI and IS has been reported for high levels of FVIII, fibrinogen, plasminogen, VWF, FX and FXIII [5]. It has also been observed that some factors associated with hypercoagulability, for example elevated FXI and FXII levels, increase the risk of IS, but not that of MI [10, 11]. Recently we showed in a study of women under 50 years of age that hypercoagulability increases the risk of IS, whereas the risk of MI is only affected marginally [12]. However, it is unclear to which extent these findings truly reflect a different role of hypercoagulability in these two diseases, or whether a difference is only present in this specific patient group, for the differential effect may be limited to specific age and sex categories [10, 11].

Differences in causal mechanisms, overall and in subgroups, are not easily recognizable because most studies only investigated one or a combination of arterial thrombosis manifestations. Some studies differentiated between MI and IS, but the results were often fragmented into several publications in different specialty journals.

If true, the hypothesis that MI and IS behave differently from a prothrombotic perspective is a strong stimulus for researches into the role of coagulation on the aetiology of IS, a field in which data are lacking compared with the equivalent of MI [13].

Therefore, we set out to identify studies that investigated markers of hypercoagulability in association with the risk of both IS and MI, in order to compare these effects directly and to test the hypothesis that hypercoagulability has a differential effect on these two main forms of arterial thrombosis.

## Methods

### Literature search and study selection

We used a systematic approach to identify study populations (with both cohort and case-control study design) in which the effect of a prothrombotic factor was studied on both MI and IS. We included in our analysis only direct comparisons within the same study population to reduce bias due to differences in study design, data acquisition, data analyses and underlying research questions. The data needed for this direct comparison were obtained by a systematic and comprehensive three-stage approach.

#### 1. Identification and selection of the publications

**Fig 1:**
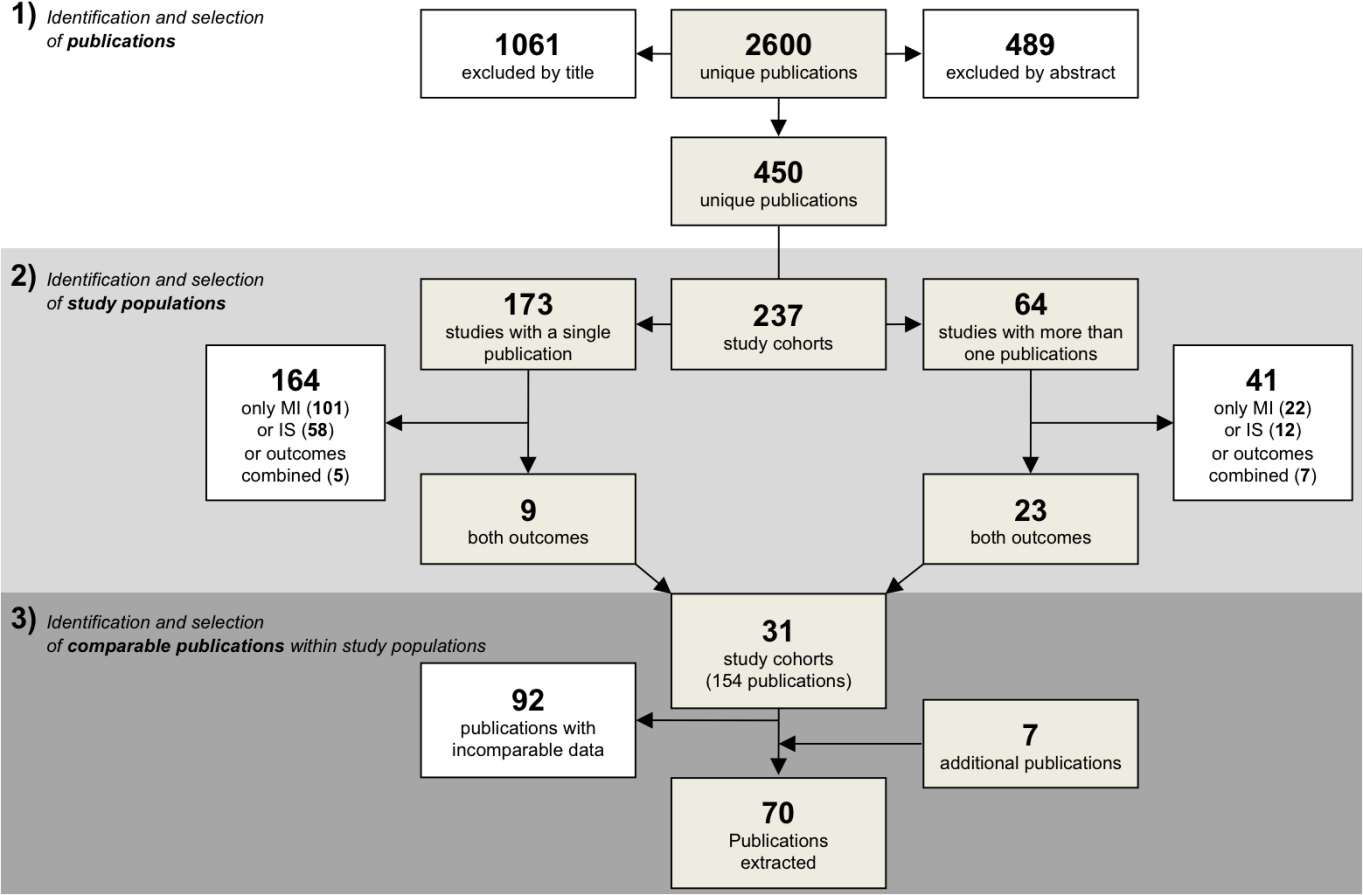
Flow chart of the steps of data collection. The figure shows the three steps in the data collection: (1) identification of publications which report on the effect of measures of hypercoagulability and the risk of myocardial infarction (MI) or ischaemic stroke (IS) (2) identification of study populations (3) identification of publications with comparable data. Comparable data can be found in the same publication or in two different publications.

We searched for all publications reporting the association between a measure of coagulation and MI or IS up to July 2012 (Fig 1; step 1). Publications were identified with a systematic search in four different search engines, PubMed (1950–2012), EMBASE (1980–2012), the Science Citation Index through Web of Science (1945–2012) and the Cochrane Library (1898–2012). The search strategy applied in each database was composed by the combination of four concepts: presence of *ischaemic stroke* or *myocardial infarction* (combined with either “AND” and “OR”) “AND” *coagulation* “AND” *risk* “AND” *cohort or case control*. These concepts were extensively searched either by the use of subject headings or free text words (S1 File). From the resulting list, publications were selected independently by two authors (AM, BS).

Publications were included when: (1) they reported original data about the association between a measure of coagulation (either coagulation factor plasma levels, activity, genetic mutation or aggregated measures) and MI or IS separately; (2) the outcomes were the clinical endpoints MI or IS as acute vascular events rather than surrogates (e.g., studies reporting only on carotid intima-media thickness or coronary artery plaque were excluded); (3) the magnitude of the association was reported as a point estimate such as odds ratio (OR), risk ratio (RR) or hazard ratio (HR), or these could be inferred from the raw data. Studies that combined several forms of stroke (e.g. transient ischaemic attack, haemorrhagic stroke, sinus thrombosis) were included as long as IS was at least part of the combined endpoint used. Review articles or previous systematic reviews were excluded but used to check for relevant publications that were not identified by the literature search.

#### 2. Identification and selection of the studies

The selected publications were then used to identify unique study populations (Fig 1; step 2). Publications were considered to be pertain of the same study population when it was clearly stated as such in the text (e.g., by study name) or when the publication shared the same group of participants, based on method description, inclusion procedures, number and baseline characteristics. Studies that included only MI or IS and therefore could not be used for a direct comparison were excluded.

#### 3. Comparison of the publications from the same study and data extraction

We selected publications from the same study populations in which the risk of a prothrombotic factor could be directly compared for MI and IS (Fig 1; step 3). This was possible when the same factor was measured in MI cases and IS cases in the same publication, or in two publications on the same study population (one on MI and one on IS) and similar analytical approaches were used for both diseases. Additionally, all reference lists were scanned to identify publications that were missed during the previous two steps. Also, key authors of each selected publication were entered in individual Pubmed searches to further identify missed publications.

Information was extracted from each selected article with a standardized form. Extracted data were: 1) study outcome (i.e. stroke of any origin, ischaemic stroke, acute myocardial infarction, angina); 2) characteristics of the marker of hypercoagulability (i.e. name, type of assay (phenotype or genotype) and the effect estimator used in the analysis); 3) study type (case-control or follow-up study); 4) magnitude of the association as a relative effect estimate (adjusted) with corresponding confidence intervals; 5) study population characteristics (i.e. number of participants, age, sex and baseline risk profile). Finally we performed a study quality assessment to assess the presence of bias that could substantially influence the results. We considered the results possibly biased if: 1) blood samples were taken in the acute phase (first month after the event), which could lead to reverse causation; 2) lack of adjustment for age and sex (for non- genetic exposures); 3) high probability of selection bias; 4) different follow-up duration between the study groups (>2 years). Studies with these characteristics were analysed separately.

Markers of hypercoagulability were categorized in markers of pro-coagulant activity, markers of anti-coagulant activity, markers of fibrinolysis and markers of platelet function and other pathways (including ADAMTS13 and von Willebrand factor).

### Statistical analysis

The relative risks for IS (RR_IS_) were compared with the relative risks for MI (RR_MI_) by calculating their ratio (RRR = RR_IS_/RR_MI_) per study with a corresponding 95% confidence intervals (CI), where the variance was based on the sum of the variances of RR_MI_ and RR_IS_. When a risk factor has a similar effect on MI and IS (either increasing the risk, decreasing the risk or no effect), the RRR equals 1, whereas an RRR>1 indicates a greater effect on IS risk than on MI, and vice versa. Each RRR refers to one single marker and, when two different studies investigated the same coagulation marker, their RRR_S_ are presented separately. To filter out small variations due to chance we used the standard error of the RRR: markers with RRRs within 1+ its own standard error (SE) and 1-SE were considered to only marginally differ and to affect the risk of IS and MI equally, whereas markers with RRRs<1-SE or RRRs>1+SE were considered to have a substantially different effect.

Subgroups were based on age (younger or older than 50 years old for women and 55 for men), sex, stroke type (only ischaemic or haemorrhagic also included), baseline risk of study population (general population or patients affected by one or more diseases with a high impact on cardiovascular risk, such as atrial fibrillation, end stage renal disease or previous cardiovascular events), type of the investigated marker (phenotypic or genotypic), study design of the original publication (case-control or follow-up), and probability of bias (high or low).

Design and results of this systematic review are reported according to the Preferred Reporting Items for Systematic Reviews and Meta-Analyses (PRISMA statement) [14].

## Results

### Literature search and studies selection

The first step of the search procedure yielded a total of 2600 publications (Fig 1). Review of titles and abstracts identified 450 potentially relevant publications. With full text reading, publications from the same study group were clustered in 237 study populations (data from 64 study populations were used in more than one publication and 173 were single publication). Thirty-one of these study populations, accounting for 154 publications, included reports on effect sizes on both MI and IS separately. S1 Table shows the characteristics of these 31 study populations. Finally, 70 publications from these study populations reported comparable measures, and were eligible for data extraction.

### Markers of hypercoagulability

**Fig 2:**
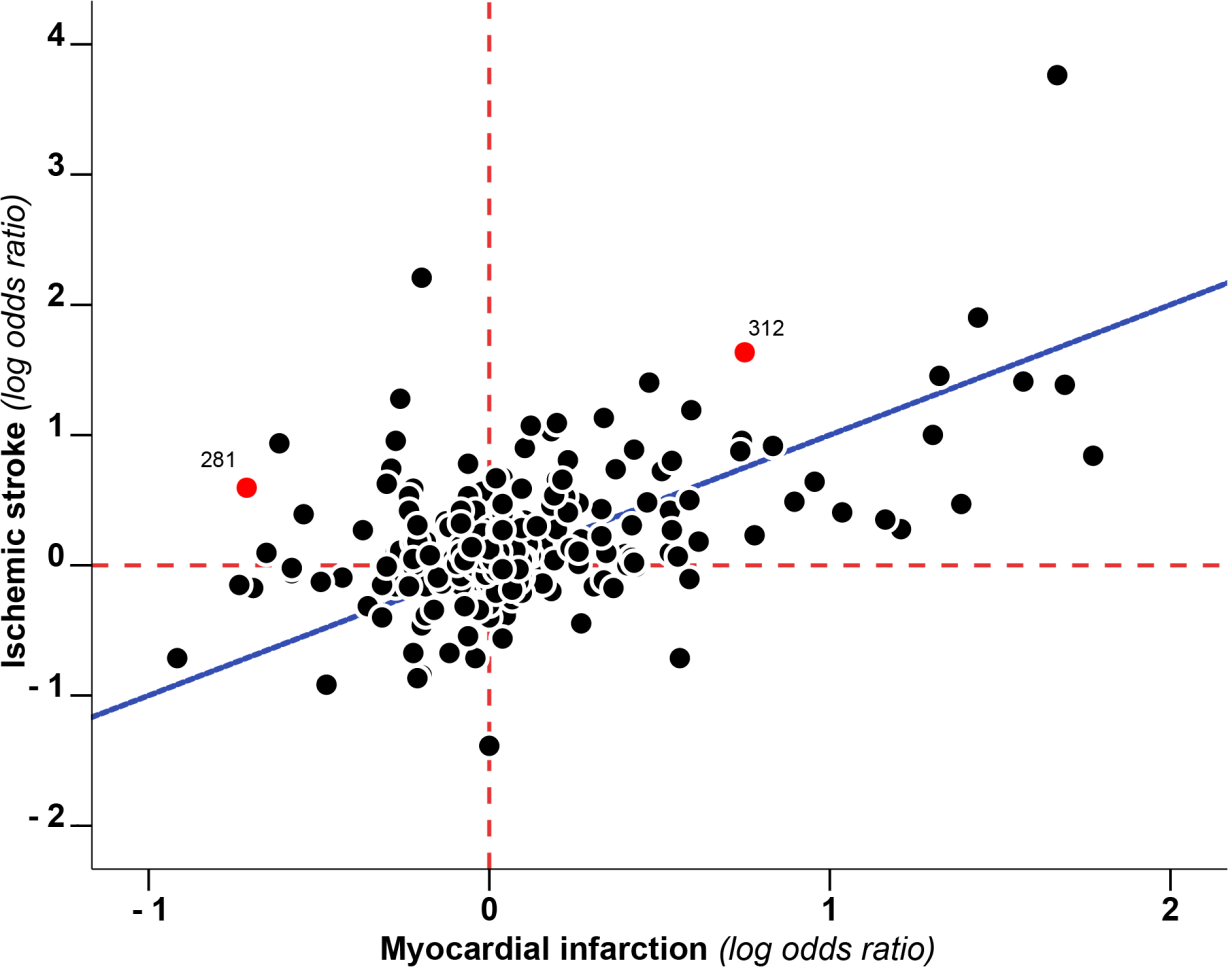
Prothrombotic risk factors and their effect on myocardial infarction and ischemic stroke. Each point depicts the log odds ratio as a measure of effect of a particular risk factor on the risk of myocardial infarction (x-axis) as well as the effect on the risk of ischaemic stroke (y-axis). The red dashed lines indicate the null effect for either myocardial infarction (vertical line) or ischaemic stroke (horizontal line). The blue diagonal line represents the theoretical line along which all points would cluster when the role of thrombotic factors is similar in the aetiology of myocardial infarction and ischaemic stroke. *As an explicative example red dots represent*: *#312*: *KAL-C1-INH (RR*_*IS*_ *5*.*14 e RR*_*MI*_ *2*.*12)*. *#281*: *FXIIIA SNP rs3024462 allele (RR*_*IS*_ *1*.*82 e RR*_*MI*_ *0*.*49)*.

**Fig 3:**
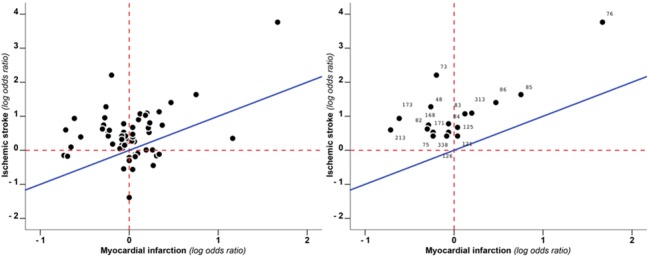
Prothrombotic risk factors with RRR either >1+SE and <1-SE (left) and either > 1+SE and <1-SE (right). Each point depicts the log odds ratio as a measure of effect of a particular risk factor on the risk of myocardial infarction (x-axis) as well as the effect on the risk of ischaemic stroke (y-axis). The red dashed lines indicate the null effect for either myocardial infarction (vertical line) or ischaemic stroke (horizontal line). The blue diagonal line represents the theoretical line along which all points would cluster when the role of thrombotic factors is similar in the aetiology of myocardial infarction and ischaemic stroke. On the left are depicted RRR>1+SE and RRR<1-SE. on the right RRR>1+2SE. No factors had RRR<1-2SE. Numbers represent the ID of the corresponding marker in Table 1 and S2, S3 and S4 Tables.

A total of 351 markers of hypercoagulability were extracted. 203 (203/351, 58%) of those had an RRR>1, 140 (140/351, 40%) <1 and 8 (8/351, 2%) = 1 (Fig 2, S1 Fig and S1, S2, S3 and S4 Tables for the detailed list). 205 of these markers involved pro-coagulant factors, 46 anti-coagulant factors, 63 markers of fibrinolysis and 37 markers of platelet function and other pathways. For 8 markers SE was not calculable due to lack of data. Of the remaining 343 markers, 277 (81%) had an RRR between 1-1SE and 1+1SE, indicating no large difference in the effect on the risk of IS and MI. Half of these markers (150/277, 54% for MI and 126/27, 46% for IS) did not show an effect on risk of either of the outcomes (0.9>RR_MI_>1.1 and 0.9>RR_IS_>1.1, S1 Fig). Of the 66 markers that were associated with either MI or IS, 49 (14% of all, 74% of those with an effect) had an RRR greater than 1+1SE, indicating a larger effect on IS risk than MI risk. Of these, 18/49 (37%) had an RRR greater than 1+2SE. The RRR of 17 (5% of all 343, 26% of those with an effect) markers of hypercoagulability was <1-1SE. There were no markers with an RRR<1-2SE (Table 1 and Fig 3). Pro-coagulant factors contributed for the greatest part to the difference between RRRs (pro-coagulant factors with RRR>1+1SE 30/199 (15%) and with RRR<1-1SE 6/199 (3%); anti-coagulant factors with RRR>1+1SE 4/45 (9%) and with RRR<1-1SE 4/45 (9%); factors involved in fibrinolysis with RRR>1+1SE 7/63 (11%) and with RRR<1-1SE 4/63 (6%); others factors with RRR>1+1SE 8/36 (22%) and with RRR<1-1SE 3/36 (8%)). Within pro-coagulant factors the largest RRRs, indicative of a larger effect on IS than on MI, were observed for FV Leiden mutation (RRR 3.42, 95% CI 0.11–104), a genetic variant in the gene coding for coagulation factor VIII (F8 rs6655259, RRR 4.72, 95% CI 0.62–35.73), presence of lupus anti-coagulant (RRR 8.13, 95% CI 0.61–108.76) and three variants of FXIII (F13A1 V34L, RRR 4.66, 95% CI 0.44–49.10; F13A1 T204P, RRR 11.1, 95% CI 5.64–21.82 and F13A1 rs3024462, RRR 3.71, 95% CI 0.62–22.35).

**Table 1:** Factors that showed a predominant association with ischaemic stroke or myocardial infarction (RRR>1+SE and RRR<1-SE).

	RRR>1+SE		RRR<1-SE		
ID	Coagulation factor (contrast)	RRR (95% CI)	ID	Coagulation factor (contrast)	RRR (95% CI)
	**Pro-coagulant**				
317	FXIIIA SNP Tyr204phe (dominant) *	11.1 (5.64–21.82)	53	fibrinopeptide A (T3 vs T1)	0.63 (0.31–1.26)
282	FVIII SNP 165293 rs6655259 (allele) *	4.72 (0.62–35.73)	301	FXIIIA SNP Pro564Leu (dominant)	0.64 (0.31–1.29)
331	FXIIIA SNP Val34Leu (L/L vs V/V) *	4.66 (0.44–49.1)	121	FGA 3807 (allele)	0.77 (0.52–1.12)
281	FXIIIA SNP 177424 (allele) *	3.71 (0.62–22.35)	123	FX SNP 9501 (allele)	0.77 (0.51–1.17)
294	FV Leiden (dominant)	3.42 (0.11–104)	284	fibrinogen (SD)	0.78 (0.61–0.98)
280	FVIII SNP 25167 (allele) *	2.8 (0.7–11.2)	128	FXI SNP 3450 (allele)	0.81 (0.59–1.11)
315	FXIa-C1-INH (>90 percentile)*	2.58 (0.77–8.72)			
313	FXIIa-C1-INH (>90 percentile) *	2.53 (0.74–8.6)			
105	FV Leiden (allele) *	2.44 (0.6–10)			
310	FXIa-AT-INH (>90 percentile) *	2.32 (0.68–7.95)			
104	FV SNP Rs7542281 (allele)	2.22 (0.65–7.56)			
307	FXIIIB SNP His95Arg (dominant) *	2.15 (0.88–5.25)			
64	d-dimer (SD (log scale)) *	1.88 (0.81–4.4)			
278	FVIII SNP 95826 (allele) *	1.81 (1.02–3.2)			
102	FV SNP Rs6035 (allele)	1.74 (0.56–5.4)			
277	FXI SNP 4197 (allele)	1.58 (0.61–4.1)			
276	FVIII SNP 55941 (allele)	1.5 (0.9–2.5)			
275	FV SNP upper 38592 (allele)	1.49 (0.69–3.21)			
63	fibrinogen (SD (log scale)) *	1.46 (0.96–2.22)			
274	FVIII SNP 139972 (allele)	1.45 (0.68–3.08)			
37	d-dimer (T3 vs T1)	1.44 (0.63–3.32)			
100	FV SNP Rs3753305 (allele)	1.39 (0.67–2.86)			
347	trombin generation (PEAK) (SD)	1.27 (0.83–1.95)			
269	FXIIIA SNP 4377 (allele)	1.26 (0.93–1.71)			
268	FX SNP 4544 (allele)	1.25 (0.83–1.87)			
267	FGA 5498 (allele)	1.25 (0.84–1.86)			
265	FXI SNP 10942 (allele)	1.22 (0.83–1.78)			
264	FV SNP lower 29565 (allele)	1.21 (0.87–1.69)			
262	TFPI SNP 2418 (allele)	1.17 (0.9–1.51)			
260	FGA 251 (allele)	1.15 (0.87–1.52)			
	**Anticoagulant**				
103	prot C SNP Rs2069920 (allele)	1.92 (0.93–3.96)	71	thombomodulin SNP Rs3176123	0.62 (0.3–1.28)
13	prot C (Q1 vs Q5) *	1.65 (1.05–2.6)	118	prot C receptor SNP 837 (allele)	0.74 (0.46–1.2)
101	prot C SNP Rs1401296 (allele) *	1.42 (0.67–3.03)	127	thombomodulin SNP 6235 (allele)	0.81 (0.58–1.13)
266	prot C SNP 11310 (allele)	1.22 (0.92–1.62)	130	prot C SNP 4515 (allele)	0.83 (0.63–1.1)
	**Fibrinolysis**				
314	CLT (hypo vs. normofibrinolysis) *	2.54 (0.71–9.09)	350	t-PA (Q4 vs Q1)	0.44 (0.17–1.15)
329	PAI-1 SNP 4G/5G (4G/5G vs 4G/4G)	2.12 (0.51–8.69)	125	t-PA SNP 30619 (allele)	0.78 (0.51–1.18)
20	TAFI SNP 1040C/T (CC vs TT)	1.79 (0.45–7.11)	129	plasminogen SNP 18114 (allele)	0.83 (0.63–1.11)
326	PAI-1 SNP 4G/5G (allele)	1.68 (0.45–6.24)	131	TAFI SNP 54691 (allele)	0.83 (0.61–1.12)
341	t-PA (SD)	1.54 (0.55–4.34)			
22	t-PA (T3 vs T1)	1.5 (0.69–3.27)			
62	t-PA (SD (log scale))	1.35 (0.81–2.25)			
	**Other**				
316	lupus anticoagulant (ratio > = 1.15)^1^ *	8.13 (0.61–108)	24	whole blood aggregation (Q5 vs Q1)	0.25 (0.07–0.93)
330	GPIb SNP thr/Met (recessive)	2.55 (0.48–13.69)	25	PLT aggregation (first) (Q5 vs Q1)	0.49 (0.18–1.31)
312	KAL-C1-INH (>90 percentile) *	2.42 (0.77–7.64)	70	ICAM1 SNP Rs3093030 (allele)	0.55 (0.26–1.17)
311	anti-beta2GP (>95 percentile)	2.33 (0.63–8.71)			
309	anti-prothrombin IgG (>95 percentile)	2.25 (0.38–13.5)			
308	ADAMTS-13 (Q1 vs Q4)	2.21 (0.65–7.51)			
327	GPIa SNP C807T (recessive)	1.78 (0.45–7.04)			
23	VWF (T3 vs T1)	1.56 (0.72–3.34)			

(^1^) Normalised ratios for LA-screen and LA-confirm coagulation times. The positivity for lupus anticoagulant was considered when the ratio was 1.15 or higher, on the basis of the 99th percentile of the value recorded for 40 healthy volunteers. More details can be found in the original publication.

(*) Prothrombotic factors with an RRR greater than 1+2SE. No prothrombotic factor had an RRR less than 1-2SE.

### Subgroups

Table 2 shows the results of the subgroup analyses. The largest RRRs were found in young individuals (RRR>1+1SE: 17/43 (40%); RRR>1+2SE: 9/17 (53%); 5 studies). Of those RRRs, 33/43 (77%) belonged to populations of only women. However, 20 RRRs out of 43 (46%) come from a single study population (the RATIO study). After the exclusion of this study, the RRRs greater than 1+1SE became 6 (6/23, 26%) (data not shown). A substantial part of the studies (15 studies, 216 factors) combined haemorrhagic and ischaemic stroke as a single outcome. When restricted to those that excluded haemorrhagic stroke, 26 out of 130 markers (20%) had an RRR >1+1SE (RRR>1+2SE 33/84 (39%)) and 6 (6/130, 4%) an RRR <1-1SE. Larger RRRs were also found in populations at a relatively low risk of arterial thrombosis (RRR>1+1SE 29/157, 20%), and for phenotypic measurements (RRR>1+1SE 18/85, 21%), whereas only 15 RRRs were potentially affected by high risk of bias and their exclusion did not change the results. A graphical representation of relevant RRRs by subgroups is shown in S2 Fig.

**Table 2:** Distribution of RRRs greater than 1+1SE and smaller than 1-1SE for different subgroups of population.

Subgroups	Prothrombotic markers	>1+1SE N (%)	>1-1SE N (%)
**Sex**			
Male	32	3 (10)	2 (6)
Female	38	16 (42)	2 (5)
No distinction	273	30 (11)	13 (5)
**Age at onset** ^1^			
Young	43	17 (40)	1 (2)
Old	300	32 (11)	16 (5)
**Ischaemic stroke type**			
Only ischaemic	130	26 (20)	6 (5)
Ischemic and haemorrhagic	213	23 (11)	11 (5)
**Cardiovascular risk** ^2^			
High	186	20 (11)	9 (5)
Low	157	29 (19)	8 (5)
**Bias risk**			
Low	328	46 (14)	17 (5)
High	15	2 (13)	0 (0)
**Type of marker**			
Phenotypic	85	18 (21)	5 (6)
Genotypic	258	31 (12)	12 (5)
**Study design**			
Case-control	222	34 (15)	10 (5)
Follow-up	121	15 (12)	7 (6)

(^1^) Young age at onset is defined as younger than 50 years old for women and 55 for men.

(^2^) Low risk for arterial thrombosis is defined as a risk comparable with the general population. High risk for arterial thrombosis is given to populations affected by one or more diseases with a high impact on cardiovascular risk (such as atrial fibrillation, end stage renal disease, previous cardiovascular event).

## Discussion

We investigated whether markers of hypercoagulability have a differential role on the risk of MI and IS. This systematic review indicates that overall hypercoagulability has a larger effect on the risk of IS than on the risk of MI (14% of the 343 markers studied had an RRR>1+1SE compared with 5% of markers with RRR<1-1SE). The majority of the markers included in this study were pro-coagulant factors, in which the difference between RRRs was remarkable (15% of these factors had an RRR>1+1SE compared with only 3% with an RRR<1-1SE), whereas no difference was found for anti-coagulant factors and a small difference for factors involved in the fibrinolytic system. The differential role was more pronounced in young patients (40% of factors with RRR>1+1SE) and after the exclusion of studies that used haemorrhagic stroke and IS as a combined endpoint (20% of factors with RRR>1+1SE). Studies with young populations were only 5 and one of those (the RATIO study) accounted for half of the RRRs. However, after the exclusion of the RATIO study from the analysis, the percentage of large RRRs remained higher than that in the other subgroups (26% of factors with RRR>1+1SE). Our study is the first that has systematically summarized the data available on the relationship between hypercoagulability and the two main manifestations of arterial thrombosis. These data support the hypothesis that hypercoagulability increases the risk of IS more than that of MI.

Ischaemic stroke is a heterogeneous disease in which several causal mechanisms play a role. According to the TOAST classification [15], subtypes of IS can be divided in five main categories, i.e., stroke from cardioembolic origin, large vessel atherosclerosis, small vessel occlusion, stroke of other determined origin and stroke of undetermined origin. All these categories have specific risk factors, such as for example atrial fibrillation for cardioembolic stroke, whereas stroke of undetermined origin has none. Stroke of undetermined origin includes a third of all strokes and half of the strokes in the young [16–18]. When the analyses were restricted to the young, the difference between MI and IS was more marked (40% of the factors had RRR greater than 1+1SE). Unfortunately, no data on TOAST classification were available in the included studies; however, we can hypothesize that the larger difference found in the young is associated with the higher incidence of stroke of undetermined origin in these patients. Notably, increasing evidence suggests that most strokes of undetermined origin are caused by covert thromboembolic events [19]. In our study we found that, whereas factors associated with platelet activation are similarly involved in the two diseases, many markers of abnormal secondary haemostasis, such as for example FV Leiden or the presence of Lupus anticoagulant, have a greater role in the risk of IS than that of MI. These markers are known risk factors for venous thrombosis, and this supports the hypothesis that, although paroxysmal atrial fibrillation and subsequent embolization of a thrombus is undoubtedly responsible for a fraction of strokes of undetermined origin, hypercoagulability in itself should also be considered in cryptogenic strokes. Unfortunately, the lack of a disease classification in the available literature prevents the possibility to further investigate this hypothesis, and underlines the need of new studies in etiologic research, particularly for IS [13, 20].

Some methodological issues should be considered. First, due to our within study population approach we could only include a small part of the available data on measures of coagulation and the risk of arterial thrombosis. This approach reduced the power of the study, but it is unlikely that this selection can explain our findings. Even more, our method increased the reliability of the results since it removes publication bias and bias introduced by differences in data collection, laboratory analysis, quality of data and statistical analysis between different study populations. Moreover, even if a bias was present in an original study, it is likely to have had a similar effect on the MI and IS analyses, leaving the RRR estimates unaffected (except for the null effects). A subgroup analysis restricted to studies with low evidence of bias yielded results similar to the overall findings, thereby indicating that there is a high level of similarity between the RR_MI_ and RR_IS_ when assessing the RRR. Finally, when the analysis was restricted to follow-up studies, results did not change substantially, indicating that reverse causation cannot explain the observed difference between MI and IS. A second limitation is the lack of a standard measure of precision of the RRR estimates. As a measure of variance, i.e. of standard error, we adopted the sum of the variance of the original IS estimate plus the MI estimate. This method is probably an overestimation of the true variance, because the two risk ratios included in the RRR share, at least partly, the same population, and therefore our approach may be considered conservative. This yielded confidence intervals of RRRs larger than the true ones, hence we arbitrarily predefined that RRRs 1±1SE reflected a true difference in the effect on MI and IS risk.

Third, MI and IS have approximately the same incidence in the general population; however, this is not the case in specific subgroups, for example, in patients with atrial fibrillation [2]. Unfortunately we were unable to adjust our estimates for the presence of such factors. However, when we limited our analyses to studies that included only low risk study populations, we found that the difference between risk factors for the two diseases was even more pronounced (19% of the RRR greater than 1+1SE, 157 factors, 25 study populations).

To identify new treatments to prevent arterial thrombosis, it is important that reliable data on potential risk factors are available. In literature, data on risk factors for IS are lacking compared with the equivalent data for MI, and there are very few data on specific subtypes of IS [13]. Our findings, supporting the hypothesis that IS and MI behave differently from a prothrombotic perspective, are a warning for the need of new investigations on the role of coagulation in subtypes of IS, especially in the young. The presence of coagulation markers that are risk factors specifically for IS and not for MI can have implications in the medical treatment of IS, especially in the era of the new direct oral anti-coagulants, drugs that are specific for a single coagulation factor (thrombin for dabigatran, activated FX for apixaban, edoxaban and rivaroxaban, and other direct inhibitors against FXI and FXII are in pre-clinical phase studies) [21, 22]. Because of differences in the role of hypercoagulability in MI and IS, their efficacy in the prevention of the two main forms of arterial thrombosis might differ.

## Supporting Information

S1 Fig Schematic representation of all the relative risk ratios (RRR) of the markers of hypercoagulability. The bars indicate the RRR for each marker of prothrombotic state. Scale is logarithmic. RRR>0 (right) greater effect on ischaemic stroke; RRR<0 (left) greater effect on myocardial infarction. 135 out of 351 markers (38%) had an RRR between 0.9 and 1.1 (red dashed lines).
(TIF)

S2 Fig Prothrombotic risk factors and their effect on myocardial infarction and ischaemic stroke by subgroups. Each point depicts the log odds ratio as a measure of effect of a particular risk factor on the risk of myocardial infarction (x-axis) as well as the effect on the risk of ischaemic stroke (y-axis). The red dashed lines indicate the null effect for either myocardial infarction (vertical line) or ischaemic stroke (horizontal line). The blue diagonal line represents the theoretical line along which all points would cluster when the role of thrombotic factors is similar in the aetiology of myocardial infarction and ischaemic stroke.
(TIF)

S1 File Search strategies. (PDF)

S1 Table Characteristics of the 31 study populations. (PDF)

S2 Table Phenotypic measurements sorted alphabetically. (PDF)

S3 Table Genotypic measurements sorted alphabetically. (PDF)

S4 Table All prothrombotic factors sorted by ID. (PDF)
