# Supplementary figures and images for: Hypercoagulability Is a Stronger Risk Factor for Ischaemic Stroke than for Myocardial Infarction: A Systematic Review
Source: PLoS One. 2015 Aug 7;10(8):e0133523. doi: 10.1371/journal.pone.0133523 (PMC4529149; doi:10.1371/journal.pone.0133523)

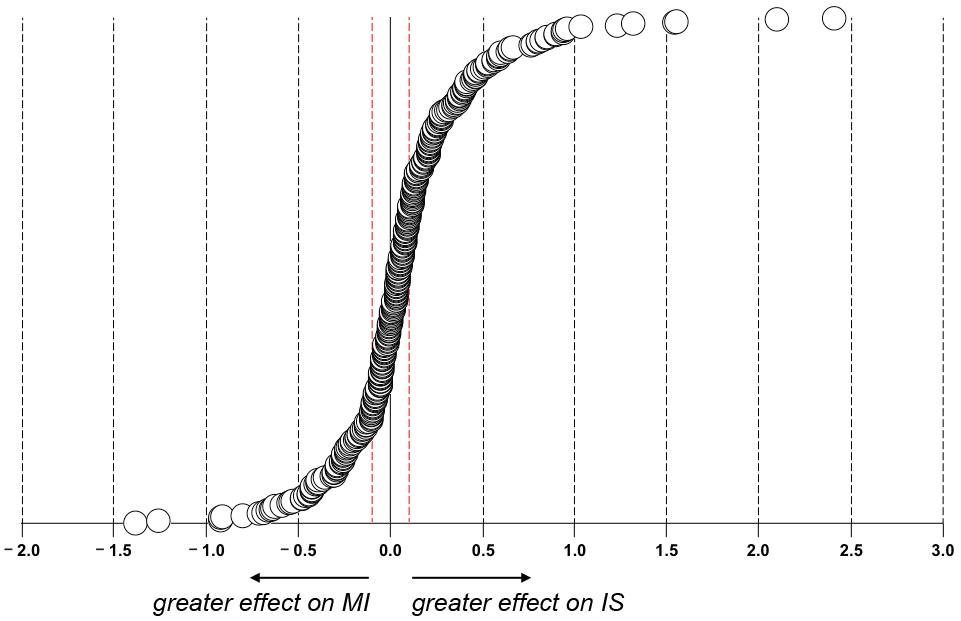

Supplement: S1 Fig — The bars indicate the RRR for each marker of prothrombotic state. Scale is logarithmic. RRR>0 (right) greater effect on ischaemic stroke; RRR<0 (left) greater effect on myocardial infarction. 135 out of 351 markers (38%) had an RRR between 0.9 and 1.1 (red dashed lines). (TIF) [file pone.0133523.s001.tif]

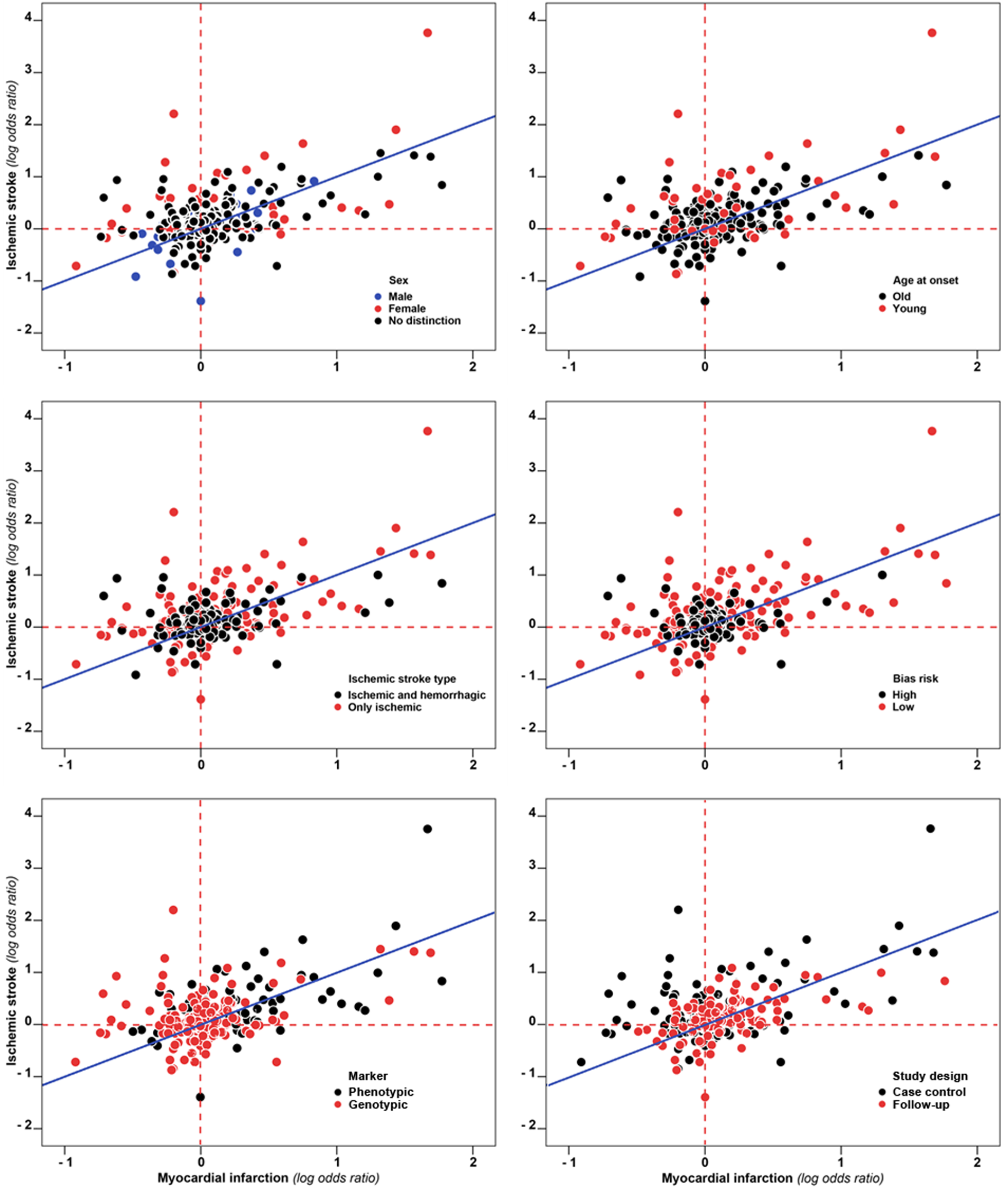

Supplement: S2 Fig — Each point depicts the log odds ratio as a measure of effect of a particular risk factor on the risk of myocardial infarction (x-axis) as well as the effect on the risk of ischaemic stroke (y-axis). The red dashed lines indicate the null effect for either myocardial infarction (vertical line) or ischaemic stroke (horizontal line). The blue diagonal line represents the theoretical line along which all points would cluster when the role of thrombotic factors is similar in the aetiology of myocardial infarction and ischaemic stroke. (TIF) [file pone.0133523.s002.tif]
