# Supplementary material for: Hypercoagulability Is a Stronger Risk Factor for Ischaemic Stroke than for Myocardial Infarction: A Systematic Review
Source: PLoS One. 2015 Aug 7;10(8):e0133523. doi: 10.1371/journal.pone.0133523 (PMC4529149; doi:10.1371/journal.pone.0133523)
Supplement: S1 File — (PDF) [file pone.0133523.s003.pdf]

S1 File. Search strategies

PubMed

(Medical Subject Headings), Major (Major Medical Subject Headings).

((((stroke OR strokes) NOT "stroke volume") OR "Stroke"[Mesh] OR "Cerebral Stroke" OR "Cerebral Strokes" OR "Brain Vascular Accident" OR "Brain Vascular Accidents" OR "Cerebrovascular Apoplexy" OR "Cerebrovascular Stroke" OR "Cerebrovascular Strokes" OR CVA[tw] OR CVAs[tw] OR Apoplexy[tw] OR "Cerebrovascular Accident" OR "Cerebrovascular Accidents" OR "Acute Stroke" OR "Acute Strokes") AND ("Myocardial Infarction"[Mesh] OR "myocardial infarction" OR "Myocardial Infarctions" OR "Myocardial Infarct" OR "Myocardial Infarcts" OR "Myocardial Ischemia"[mesh] OR "Cardiovascular Diseases"[Mesh:NoExp] OR "coronary heart disease" OR "coronary disease") AND (coagulation OR "Blood Coagulation"[Mesh] OR "Hemostasis"[Mesh:NoExp] OR "blood clotting" OR "Blood Coagulation Factors"[Mesh] OR Blood Coagulation Tests OR Coagulants OR Hemostatics OR Partial Thromboplastin Time OR Prothrombin Time OR Thrombin Time OR Whole Blood Coagulation Time OR coagulant OR fibrinogen OR fibrinogens OR fibrinolysis) AND (risk OR risks OR "Risk factors"[mesh] OR "Risk"[mesh]) AND ("Cohort Studies"[mesh] OR cohort OR cohorts OR followup OR "follow up" OR "Epidemiologic Studies"[mesh] OR "Case-Control Studies"[mesh] OR casecontrol OR "case-control" OR "case-controlled" OR "Retrospective Studies"[mesh] OR "Longitudinal Studies"[mesh] OR "Cross-Sectional Studies"[mesh] OR "Prospective studies"[mesh] OR "Retrospective Studies" OR "Longitudinal Studies" OR "Cross-Sectional Studies" OR "Prospective studies" OR "Retrospective Study" OR "Longitudinal Study" OR "Cross-Sectional Study" OR "Prospective study" OR "Comparative Study"[Publication Type])) OR (((((stroke[ti] OR strokes[ti]) NOT "stroke volume") OR "Stroke"[Majr] OR "Cerebral Stroke"[ti] OR "Cerebral Strokes"[ti] OR "Cerebrovascular Apoplexy"[ti] OR "Cerebrovascular Stroke"[ti] OR "Cerebrovascular Strokes"[ti] OR CVA[ti] OR CVAs[ti] OR Apoplexy[ti] OR "Cerebrovascular Accident"[ti] OR "Cerebrovascular Accidents"[ti] OR "Acute Stroke"[ti] OR "Acute Strokes"[ti]) OR ("Myocardial Infarction"[Majr] OR "myocardial infarction"[ti] OR "Myocardial Infarctions"[ti] OR "Myocardial Infarct"[ti] OR "Myocardial Infarcts"[ti] OR "Myocardial Ischemia"[majr] OR "Cardiovascular Diseases"[Majr:NoExp])) AND (coagulation[ti] OR "Blood Coagulation"[Majr] OR "Hemostasis"[Majr:NoExp] OR "blood clotting"[ti] OR "Blood Coagulation Factors"[Majr] OR Coagulants[ti] OR Hemostatics[ti] OR Hemostatic[ti] OR Haemostatics[ti] OR Haemostatic[ti] OR Haemostasis[ti] OR Hemostasis[ti] OR Partial Thromboplastin Time[ti] OR Prothrombin Time[ti] OR Thrombin Time[ti] OR coagulant[ti] OR fibrinogen[ti] OR fibrinogens[ti] OR fibrinolysis[ti]) AND (risk OR risks OR "Risk factors"[mesh] OR "Risk"[mesh]) AND ("Cohort Studies"[mesh] OR cohort OR cohorts OR followup OR "follow up" OR "Epidemiologic Studies"[mesh] OR "Case-Control Studies"[mesh] OR casecontrol OR "case-control" OR "case-controlled" OR "Retrospective Studies"[mesh] OR "Longitudinal Studies"[mesh] OR "Cross-Sectional Studies"[mesh] OR "Prospective studies"[mesh] OR "Retrospective Studies" OR "Longitudinal Studies" OR "Cross-Sectional Studies" OR "Prospective studies" OR

"Retrospective Study" OR "Longitudinal Study" OR "Cross-Sectional Study" OR "Prospective study" OR "Comparative Study"[Publication Type])) **AND english[la]**

EMBASE

((*Stroke/ OR *Stroke patient/ OR Stroke.ti OR "Cerebral Strokes".ti OR "Cerebrovascular Apoplexy".ti OR "Cerebrovascular Stroke".ti OR "Cerebrovascular Strokes".ti OR CVA.ti OR CVAs.ti OR Apoplexy.ti OR "Cerebrovascular Accident".ti OR "Cerebrovascular Accidents".ti OR "Acute Stroke".ti OR "Acute Strokes".ti) OR (exp *Heart Infarction/ OR *Ischemic Heart Disease/ OR *Heart Disease/ OR "myocardial infarction".ti OR "Myocardial Infarctions".ti OR "Myocardial Infarct".ti OR "Myocardial Infarcts".ti)) AND (exp *Blood clotting/ OR exp *blood clotting factor/ OR *Hemostasis/ OR coagulation.ti OR "blood clotting".ti OR Coagulants.ti OR Hemostatics.ti OR Hemostatic.ti OR Haemostatics.ti OR Haemostatic.ti OR Haemostasis.ti OR Hemostasis.ti OR Partial Thromboplastin Time.ti OR Prothrombin Time.ti OR Thrombin Time.ti OR coagulant.ti OR fibrinogen.ti OR fibrinogens.ti OR fibrinolysis.ti) AND (exp risk factor/ OR exp risk/ OR risk*.mp) AND (cohort analysis/ OR cohort*.mp OR exp follow up/ OR followup.mp OR "follow up".mp OR Case Control Study/ OR casecontrol*.mp OR "case-control*".mp OR Retrospective Study/ OR Longitudinal Study/ OR Cross-Sectional Study/ OR Prospective study/ OR ("Retrospective Studies" OR "Longitudinal Studies" OR "Cross-Sectional Studies" OR "Prospective studies" OR "Retrospective Study" OR "Longitudinal Study" OR "Cross-Sectional Study" OR "Prospective study" OR "Comparative Study" OR "Comparative studies").mp OR Comparative Study/)

Web of Science

TI=((Stroke OR "Cerebral Strokes" OR "Cerebrovascular Apoplexy" OR "Cerebrovascular Stroke" OR "Cerebrovascular Strokes" OR CVA OR CVAs OR Apoplexy OR "Cerebrovascular Accident" OR "Cerebrovascular Accidents" OR "Acute Stroke" OR "Acute Strokes") OR ("Heart Infarction" OR "Ischemic Heart Disease*" OR "Heart Disease*" OR "myocardial infarction" OR "Myocardial Infarctions" OR "Myocardial Infarct" OR "Myocardial Infarcts")) AND TI=("Blood clotting" OR Hemostasis OR coagulation OR "blood clotting" OR Coagulants OR Hemostatics OR Hemostatic OR Haemostatics OR Haemostatic OR Haemostasis OR Hemostasis OR Partial Thromboplastin Time OR Prothrombin Time OR Thrombin Time OR coagulant OR fibrinogen OR fibrinogens OR fibrinolysis) AND TS=risk* AND TS=("cohort analysis" OR "cohort stud*" OR "follow up stud*" OR "followup stud*" OR "Case Control Stud*" OR casecontrol* OR "case-control*" OR "Retrospective Stud*" OR "Longitudinal Stud*" OR "Cross-Sectional Stud*" OR "Prospective stud*" OR "Retrospective Studies" OR "Longitudinal Studies" OR "Cross-Sectional Studies" OR "Prospective studies" OR "Retrospective Study" OR "Longitudinal Study" OR "Cross-Sectional Study" OR "Prospective study" OR "Comparative Study" OR "Comparative studies" OR "Comparative Stud*")

Cochrane

ti/ab/kw

((Stroke OR "Cerebral Strokes" OR "Cerebrovascular Apoplexy" OR "Cerebrovascular Stroke" OR "Cerebrovascular Strokes" OR CVA OR CVAs OR Apoplexy OR "Cerebrovascular Accident" OR "Cerebrovascular Accidents" OR "Acute Stroke" OR "Acute Strokes") OR ("Heart Infarction" OR "Ischemic Heart Disease*" OR "myocardial infarction" OR "Myocardial Infarctions" OR "Myocardial Infarct" OR "Myocardial Infarcts"))

all text

("Blood clotting" OR Hemostasis OR coagulation OR "blood clotting" OR Coagulants OR Hemostatics OR Hemostatic OR Haemostatics OR Haemostatic OR Haemostasis OR Hemostasis OR Partial Thromboplastin Time OR Prothrombin Time OR Thrombin Time OR coagulant OR fibrinogen OR fibrinogens OR fibrinolysis) AND risk* AND ("cohort analysis" OR "cohort stud*" OR "follow up stud*" OR "followup stud*" OR "Case Control Stud*" OR casecontrol* OR "case-control*" OR "Retrospective Stud*" OR "Longitudinal Stud*" OR "Cross-Sectional Stud*" OR "Prospective stud*" OR "Retrospective Studies" OR "Longitudinal Studies" OR "Cross-Sectional Studies" OR "Prospective studies" OR "Retrospective Study" OR "Longitudinal Study" OR "Cross-Sectional Study" OR "Prospective study" OR "Comparative Study" OR "Comparative studies" OR "Comparative Study*")
