# Supplementary material for: Hypercoagulability Is a Stronger Risk Factor for Ischaemic Stroke than for Myocardial Infarction: A Systematic Review
Source: PLoS One. 2015 Aug 7;10(8):e0133523. doi: 10.1371/journal.pone.0133523 (PMC4529149; doi:10.1371/journal.pone.0133523)
Supplement: S1 Table — (PDF) [file pone.0133523.s004.pdf]

S1 Table. Characteristics of the 31 studies selected.

Name of the study[1]	Publications (N and references)	Year[2]	Study design[3]	Age[4]	Sex[5] (male%)	MI cases[6] (min-max)	IS cases[6] (min-max)	Prothrombotic markers[7]
ARIC	4 [1-3]	1987-1989	F-UP	45-64	42	368-1257	89-613	*1-14*
ATTAC	3 [4, 5]	2008	CC	18-55	37	198-271	103-150	*15-20*
British Regional Heart	2 [6, 7]	1980-1998	F-UP	40-50	100	198	187	*21-23*
Caerphilly	3 [8-10]	1979-1983	F-UP	45-59	100	54-353	26-156	*24-39*
Cardiovascular Health	4 [11-14]	1989	F-UP	> 65	39	158-494	115-442	*40-45*
Copenhagen City Heart	2 [15, 16]	1976-1978	F-UP	20-95	43	469-720	410-614	*46-51*
Dai K et al	1 [17]	2000	CC	54	50	49-103	107-150	*52*
Edinburg Artery	2 [18, 19]	1987	F-UP	55-74	52	166-248	45-168	*53-64*
Engstrom G et al	1 [20]	1973-1974	F-UP	47	100	611	238	*65*
Finrisk '92	2 [21, 22]	1992	F-UP	45-64	48	133	75	*66-69*
Finrisk '92-'97	1 [23]	1992-1997	F-UP	57	72	401	149	*70-105*
Framingham	1 [24]	1948	F-UP	47-79	44	214	92	*106,107*
Gonzalez-Conejero R et al	3 [25-27]	1998	CC	18-90	62	101	104	*108-111*
Group Health Cooperative	1 [28]	1995-2002	CC	30-79	39	856	368	*112-282*
Health ABC	1 [29]	1997-1998	F-UP	70-79	49	177	104	*283*
Health Survey for England	1 [30]	1994-2004	F-UP	55	45	102	126	*284*
Karakus Z et al	1 [31]	2005	CC	<50	60	63	22	*285, 286*
Northern Sweden Health and Disease	2 [32, 33]	1985-1999	F-UP	30-60	62	78	108	*287, 288*
Northwick park Heart II	1 [34]	1989	F-UP	50-60	100	231	56	*289-292*
Onohara T et al	1 [35]	1985-1995	F-UP	69	81	36	32	*293*
Pestana CI et al	1 [36]	2005-2007	CC	58	51	175	54	*294*
Physicians' Health	3 [37-39]	1984	F-UP	40-84	100	374-404	209-259	*295-297*
RATIO	9 [40-47]	1990-1995	CC	18-49	0	248	203	*298-317*
Roosendaal FR et al	8 [48-53]	1991-1995	CC	18-44	0	79-84	104-107	*318-331*
Rotterdam	4 [54-57]	1990	F-UP	>55	40	115-473	112-290	*332-335*
Ruan C et al	1 [58]	2003	CC	na	na	103	150	*336*
Santamaria A et al	2 [59]	1998-2003	CC	21-80	57	174	205	*337*
Smith FB et al	1 [60]	1989-1990	F-UP	65	64	160	79	*338-341*
Three-City	2 [61, 62]	1999-2001	F-UP	>65	39	88-94	87-90	*342-347*
TPT	1 [63]	1983-1992	F-UP	45-69	100	1515	391	*348, 349*
Women's Health Initiative	2 [64, 65]	1992	F-UP	50-79	0	304	972	*350, 351*

MI: myocardial infarction; IS: ischaemic stroke; na: information not available. ([1]) Name of the study; when this was not available, the name of the first author of the first publication is displayed. ([2]) Inclusion period, when this was not available, the year of the first publication is displayed. ([3]) F-UP: follow-up study cohort; CC: case-control study. ([4]) Age at inclusion; range, or cut off. If criteria were not available, the mean baseline age as displayed in the earliest publication. ([5]) Percentages of males reported in the first publication. In follow-up studies we report the percentage of male in the entire cohort; in case-control studies that in the control group. ([6]) Numbers show the minimum and maximum sample size of cases included in each study, and may vary over time or analyses. ([7]) Numbers show the ID of the marker. For the entire list of markers of prothrombotic state, refer to S2-4 Tables.

REFERENCES

1. Folsom AR, Ohira T, Yamagishi K, Cushman M. Low protein c and incidence of ischemic stroke and coronary heart disease: The atherosclerosis risk in communities (aric) study. *Journal of Thrombosis and Haemostasis*. 2009;7
2. Suri MF, Yamagishi K, Aleksic N, Hannan PJ, Folsom AR. Novel hemostatic factor levels and risk of ischemic stroke: The atherosclerosis risk in communities (aric) study. *Cerebrovasc.Dis.* 2010;29:497-502
3. Folsom AR, Aleksic N, Park E, Salomaa V, Juneja H, Wu KK. Prospective study of fibrinolytic factors and incident coronary heart disease: The atherosclerosis risk in communities (aric) study. *Arterioscler.Thromb.Vasc.Biol.* 2001;21:611-617
4. van Schie MC, de Maat MP, Isaacs A, van Duijn CM, Deckers JW, Dippel DW, et al. Variation in the von willebrand factor gene is associated with von willebrand factor levels and with the risk for cardiovascular disease. *Blood.* 2011;117:1393-1399
5. de Bruijne EL, Gils A, Guimaraes AH, Dippel DW, Deckers JW, van den Meiracker AH, et al. The role of thrombin activatable fibrinolysis inhibitor in arterial thrombosis at a young age: The attac study. *J.Thromb.Haemost.* 2009;7:919-927
6. Wannamethee SG, Whincup PH, Shaper AG, Rumley A, Lennon L, Lowe GD. Circulating inflammatory and hemostatic biomarkers are associated with risk of myocardial infarction and coronary death, but not angina pectoris, in older men. *J.Thromb.Haemost.* 2009;7:1605-1611
7. Wannamethee SG, Whincup PH, Lennon L, Rumley A, Lowe GD. Fibrin d-dimer, tissue-type plasminogen activator, von willebrand factor, and risk of incident stroke in older men. *Stroke.* 2012;43:1206-1211
8. Smith A, Patterson C, Yarnell J, Rumley A, Ben-Shlomo Y, Lowe G. Which hemostatic markers add to the predictive value of conventional risk factors for coronary heart disease and ischemic stroke? The caerphilly study. *Circulation*. 2005;112
9. Elwood PC, Beswick A, Pickering J, McCarron P, O'Brien JR, Renaud SR, et al. Platelet tests in the prediction of myocardial infarction and ischaemic stroke: Evidence from the caerphilly prospective study. *Br.J.Haematol.* 2001;113:514-520
10. Elwood PC, Pickering J, Yarnell J, O'Brien JR, Ben Shlomo Y, Bath P. Bleeding time, stroke and myocardial infarction: The caerphilly prospective study. *Platelets*. 2003;14:139-141
11. Reiner AP, Carty CL, Jenny NS, Nievergelt C, Cushman M, Stearns-Kurosawa DJ, et al. Proc, procr and pros1 polymorphisms, plasma anticoagulant phenotypes, and risk of cardiovascular disease and mortality in older adults: The cardiovascular health study. *J.Thromb.Haemost.* 2008;6:1625-1632
12. Crainich P, Jenny NS, Tang Z, Arnold AM, Kuller LH, Manolio T, et al. Lack of association of the plasminogen activator inhibitor-1 4g/5g promoter polymorphism with cardiovascular disease in the elderly. *J.Thromb.Haemost.* 2003;1:1799-1804
13. Smiles AM, Jenny NS, Tang Z, Arnold A, Cushman M, Tracy RP. No association of plasma prothrombin concentration or the g20210a mutation with incident cardiovascular disease: Results from the cardiovascular health study. *Thromb.Haemost.* 2002;87:614-621
14. Tracy RP, Arnold AM, Ettinger W, Fried L, Meilahn E, Savage P. The relationship of fibrinogen and factors vii and viii to incident cardiovascular disease and death in the elderly: Results from the cardiovascular health study. *Arterioscler.Thromb.Vasc.Biol.* 1999;19:1776-1783
15. Juul K, Tybjaerg-Hansen A, Steffensen R, Kofoed S, Jensen G, Nordestgaard BG. Factor v leiden: The copenhagen city heart study and 2 meta-analyses. *Blood.* 2002;100:3-10
16. Weischer M, Juul K, Zacho J, Jensen GB, Steffensen R, Schroeder TV, et al. Prothrombin and risk of venous thromboembolism, ischemic heart disease and ischemic cerebrovascular disease in the general population. *Atherosclerosis.* 2010;208:480-483

17. Dai K, Gao W, Ruan C. The sma i polymorphism in the von willebrand factor gene associated with acute ischemic stroke. *Thromb.Res.* 2001;104:389-395
18. Tzoulaki I, Murray GD, Lee AJ, Rumley A, Lowe GD, Fowkes FG. Relative value of inflammatory, hemostatic, and rheological factors for incident myocardial infarction and stroke: The edinburgh artery study. *Circulation.* 2007;115:2119-2127
19. Smith FB, Lee AJ, Fowkes FG, Price JF, Rumley A, Lowe GD. Hemostatic factors as predictors of ischemic heart disease and stroke in the edinburgh artery study. *Arterioscler.Thromb.Vasc.Biol.* 1997;17:3321-3325
20. Engstrom G, Lind P, Hedblad B, Stavenow L, Janzon L, Lindgarde F. Effects of cholesterol and inflammation-sensitive plasma proteins on incidence of myocardial infarction and stroke in men. *Circulation.* 2002;105:2632-2637
21. Rajecki M, Pajunen P, Jousilahti P, Rasi V, Vahtera E, Salomaa V. Hemostatic factors as predictors of stroke and cardiovascular diseases: The finrisk '92 hemostasis study. *Blood Coagul.Fibrinolysis.* 2005;16:119-124
22. Salomaa V, Rasi V, Kulathinal S, Vahtera E, Jauhiainen M, Ehnholm C, et al. Hemostatic factors as predictors of coronary events and total mortality: The finrisk '92 hemostasis study. *Arterioscler.Thromb.Vasc.Biol.* 2002;22:353-358
23. Auro K, Alanne M, Kristiansson K, Silander K, Kuulasmaa K, Salomaa V, et al. Combined effects of thrombosis pathway gene variants predict cardiovascular events. *PLoS.Genet.* 2007;3:e120
24. Kannel WB, Wolf PA, Castelli WP, D'Agostino RB. Fibrinogen and risk of cardiovascular disease. The framingham study. *D'Agostino,R.B.* 1987;258:1183-1186
25. Corral J, Gonzalez-Conejero R, Iniesta JA, Rivera J, Martinez C, Vicente V. The fxiii val34leu polymorphism in venous and arterial thromboembolism. *Haematologica.* 2000;85:293-297
26. Lozano ML, Gonzalez-Conejero R, Corral J, Rivera J, Iniesta JA, Martinez C, et al. Polymorphisms of p-selectin glycoprotein ligand-1 are associated with neutrophil-platelet adhesion and with ischaemic cerebrovascular disease. *British Journal of Haematology*. 2001;115
27. Gonzalez-Conejero R, Lozano ML, Rivera J, Corral J, Iniesta JA, Moraleda JM, et al. Polymorphisms of platelet membrane glycoprotein ib associated with arterial thrombotic disease. *Blood*. 1998;92:2771-2776
28. Smith NL, Bis JC, Biagiotti S, Rice K, Lumley T, Kooperberg C, et al. Variation in 24 hemostatic genes and associations with non-fatal myocardial infarction and ischemic stroke. *J.Thromb.Haemost.* 2008;6:45-53
29. Ding J, Nicklas BJ, Fallin MD, de RN, Kritchevsky SB, Pahor M, et al. Plasminogen activator inhibitor type 1 gene polymorphisms and haplotypes are associated with plasma plasminogen activator inhibitor type 1 levels but not with myocardial infarction or stroke. *Am.Heart J.* 2006;152:1109-1115
30. Hamer M, Batty GD, Stamatakis E, Kivimaki M. Comparison of risk factors for fatal stroke and ischemic heart disease: A prospective follow up of the health survey for england. *Atherosclerosis.* 2011;219:807-810
31. Karakus Z, Gurkan E, Baslamisli F, Tanriverdi K. Prothrombotic heritable risk factors for cerebral ischemic infarction, acute myocardial infarction and venous thrombosis in young adult turkish patients. *Annals of Medical Sciences*. 2005;14
32. Thogersen AM, Jansson JH, Boman K, Nilsson TK, Weinehall L, Huhtasaari F, et al. High plasminogen activator inhibitor and tissue plasminogen activator levels in plasma precede a first acute myocardial infarction in both men and women: Evidence for the fibrinolytic system as an independent primary risk factor. *Circulation.* 1998;98:2241-2247
33. Johansson L, Jansson JH, Boman K, Nilsson TK, Stegmayr B, Hallmans G. Tissue plasminogen activator, plasminogen activator inhibitor-1, and tissue plasminogen activator/plasminogen activator inhibitor-1 complex as risk factors for the development of a first stroke. *Stroke.* 2000;31:26-32

34. Govers-Riemslag JW, Smid M, Cooper JA, Bauer KA, Rosenberg RD, Hack CE, et al. The plasma kallikrein-kinin system and risk of cardiovascular disease in men. *J.Thromb.Haemost.* 2007;5:1896-1903
35. Onohara T, Komori K, Kume M, Ishida M, Ohta S, Takeuchi K, et al. Increased plasma fibrinogen level and future risk of coronary artery disease after repair of abdominal aortic aneurysm. *J.Am.Coll.Surg.* 2000;191:619-625
36. Pestana CI, Torres A, Blanco S, Rojas MJ, Mendez C, Lopez JL, et al. Factor v leiden and the risk of venous thrombosis, myocardial infarction, and stroke: A case-control study in venezuela. *Genet.Test.Mol.Biomarkers.* 2009;13:537-542
37. Blake GJ, Schmitz C, Lindpaintner K, Ridker PM. Mutation in the promoter region of the beta-fibrinogen gene and the risk of future myocardial infarction, stroke and venous thrombosis. *Eur.Heart J.* 2001;22:2262-2266
38. Ridker PM, Hennekens CH, Miletich JP. G20210a mutation in prothrombin gene and risk of myocardial infarction, stroke, and venous thrombosis in a large cohort of us men. *Circulation.* 1999;99:999-1004
39. Ridker PM, Hennekens CH, Lindpaintner K, Stampfer MJ, Eisenberg PR, Miletich JP. Mutation in the gene coding for coagulation factor v and the risk of myocardial infarction, stroke, and venous thrombosis in apparently healthy men. *N.Engl.J.Med.* 1995;332:912-917
40. Andersson HM, Siegerink B, Luken BM, Crawley JT, Algra A, Lane DA, et al. High vwf, low adamts13, and oral contraceptives increase the risk of ischemic stroke and myocardial infarction in young women. *Blood*. 2012;119:1555-1560
41. Siegerink B, Meltzer ME, de Groot PG, Algra A, Lisman T, Rosendaal FR. Clot lysis time and the risk of myocardial infarction and ischaemic stroke in young women; results from the ratio case-control study. *Br.J.Haematol.* 2012;156:252-258
42. Siegerink B, Govers-Riemslag JW, Rosendaal FR, Ten Cate H, Algra A. Intrinsic coagulation activation and the risk of arterial thrombosis in young women: Results from the risk of arterial thrombosis in relation to oral contraceptives (ratio) case-control study. *Circulation*. 2010;122:1854-1861
43. Siegerink B, Rosendaal FR, Algra A. Genetic variation in coagulation factor xiii and the risk of arterial thrombosis: Differences between myocardial infarction and ischaemic stroke. *Journal of Thrombosis and Haemostasis*. 2009;Conference
44. Urbanus RT, Siegerink B, Roest M, Rosendaal FR, de Groot PG, Algra A. Antiphospholipid antibodies and risk of myocardial infarction and ischaemic stroke in young women in the ratio study: A case-control study. *Lancet Neurol.* 2009;8:998-1005
45. Pruissen DM, Slooter AJ, Rosendaal FR, van der Graaf Y, Algra A. Coagulation factor xiii gene variation, oral contraceptives, and risk of ischemic stroke. *Blood.* 2008;111:1282-1286
46. Tanis BC, Bloemenkamp DG, van den Bosch MA, Kemmeren JM, Algra A, van de Graaf Y, et al. Prothrombotic coagulation defects and cardiovascular risk factors in young women with acute myocardial infarction. *Br.J.Haematol.* 2003;122:471-478
47. Slooter AJ, Rosendaal FR, Tanis BC, Kemmeren JM, van der Graaf Y, Algra A. Prothrombotic conditions, oral contraceptives, and the risk of ischemic stroke. *J Thromb Haemost*. 2005;3:1213-1217
48. Hindorff LA, Schwartz SM, Siscovick DS, Psaty BM, Longstreth WT, Jr., Reiner AP. The association of pai-1 promoter 4g/5g insertion/deletion polymorphism with myocardial infarction and stroke in young women. *J.Cardiovasc.Risk.* 2002;9:131-137
49. Longstreth WT, Jr., Rosendaal FR, Siscovick DS, Vos HL, Schwartz SM, Psaty BM, et al. Risk of stroke in young women and two prothrombotic mutations: Factor v leiden and prothrombin gene variant (g20210a). *Stroke.* 1998;29:577-580
50. Rosendaal FR, Siscovick DS, Schwartz SM, Psaty BM, Raghunathan TE, Vos HL. A common prothrombin variant (20210 g to a) increases the risk of myocardial infarction in young women. *Blood.* 1997;90:1747-1750

51. Reiner AP, Frank MB, Schwartz SM, Linenberger ML, Longstreth WT, Teramura G, et al. Coagulation factor xiii polymorphisms and the risk of myocardial infarction and ischaemic stroke in young women. *Br.J.Haematol.* 2002;116:376-382
52. Reiner AP, Kumar PN, Schwartz SM, Longstreth WT, Jr., Pearce RM, Rosendaal FR, et al. Genetic variants of platelet glycoprotein receptors and risk of stroke in young women. *Stroke*. 2000;31:1628-1633
53. Reiner AP, Schwartz SM, Kumar PN, Rosendaal FR, Pearce RM, Aramaki KM, et al. Platelet glycoprotein iib polymorphism, traditional risk factors and non-fatal myocardial infarction in young women. *Br J Haematol*. 2001;112:632-636
54. van der Bom JG, Bots ML, Haverkate F, Slagboom PE, Meijer P, de Jong PT, et al. Reduced response to activated protein c is associated with increased risk for cerebrovascular disease. *Ann.Intern.Med.* 1996;125:265-269
55. van Loon JE, De Maat MPM, Hofman A, Witteman JCM, Leebeek FWG. Relationship between thrombospondin gene variations, von willebrand factor levels and the risk of coronary heart disease in an older population. *Journal of Thrombosis and Haemostasis*. 2011;9
56. Wieberdink RG, van Schie MC, Koudstaal PJ, Hofman A, Witteman JC, de Maat MP, et al. High von willebrand factor levels increase the risk of stroke: The rotterdam study. *Stroke.* 2010;41:2151-2156
57. van der Bom JG, Bots ML, Haverkate F, Meijer P, Hofman A, Kluft C, et al. Activation products of the haemostatic system in coronary, cerebrovascular and peripheral arterial disease. *Thromb Haemost*. 2001;85:234-239
58. Ruan C, Dai L, Su J, Wang Z, Ruan C. The frequency of p475s polymorphism in von willebrand factor-cleaving protease in the chinese population and its relevance to arterial thrombotic disorders. *Thromb Haemost*. 2004;91:1257-1258
59. Santamaria A, Mateo J, Tirado I, Oliver A, Belvis R, Marti-Fabregas J, et al. Homozygosity of the t allele of the 46 c->t polymorphism in the f12 gene is a risk factor for ischemic stroke in the spanish population. *Stroke.* 2004;35:1795-1799
60. Smith FB, Rumley A, Lee AJ, Leng GC, Fowkes FG, Lowe GD. Haemostatic factors and prediction of ischaemic heart disease and stroke in claudicants. *Br.J.Haematol.* 1998;100:758-763
61. Carcaillon L, Alhenc-Gelas M, Bejot Y, Spaft C, Ducimetiere P, Ritchie K, et al. Increased thrombin generation is associated with acute ischemic stroke but not with coronary heart disease in the elderly: The three-city cohort study. *Arterioscler.Thromb.Vasc.Biol.* 2011;31:1445-1451
62. Carcaillon L, Gaussem P, Ducimetiere P, Giroud M, Ritchie K, Dartigues JF, et al. Elevated plasma fibrin d-dimer as a risk factor for vascular dementia: The three-city cohort study. *J.Thromb.Haemost.* 2009;7:1972-1978
63. Rudnicka AR, Mt-Isa S, Meade TW. Associations of plasma fibrinogen and factor vii clotting activity with coronary heart disease and stroke: Prospective cohort study from the screening phase of the thrombosis prevention trial. *J.Thromb.Haemost.* 2006;4:2405-2410
64. Kaplan RC, McGinn AP, Baird AE, Hendrix SL, Kooperberg C, Lynch J, et al. Inflammation and hemostasis biomarkers for predicting stroke in postmenopausal women: The women's health initiative observational study. *J.Stroke Cerebrovasc.Dis.* 2008;17:344-355
65. Pradhan AD, LaCroix AZ, Langer RD, Trevisan M, Lewis CE, Hsia JA, et al. Tissue plasminogen activator antigen and d-dimer as markers for atherothrombotic risk among healthy postmenopausal women. *Circulation.* 2004;20;110:292-300
