# Supplementary material for: Hypercoagulability Is a Stronger Risk Factor for Ischaemic Stroke than for Myocardial Infarction: A Systematic Review
Source: PLoS One. 2015 Aug 7;10(8):e0133523. doi: 10.1371/journal.pone.0133523 (PMC4529149; doi:10.1371/journal.pone.0133523)
Supplement: S2 Table — (PDF) [file pone.0133523.s005.pdf]

S2 Table. Phenotypic measurements sorted alphabetically.

ID	Factor (contrast)	RR MI	RR IS	RRR (95% CI)
308	ADAMTS13 (Q1 vs Q4)	1.40	3.10	2.21 (0.65 - 7.51)
24	aggregation (whole blood) (Q5 vs Q1)	1.00	0.25	0.25 (0.07 - 0.93)
14	alpha2 antiplasmin (Q4 vs Q1)	0.81	1.36	1.68 (0.39 - 7.21)
311	anti-beta2GP (>95 percentile)	1.20	2.80	2.33 (0.63 - 8.71)
298	anti-cardiolipin IgG (>95 percentile)	1.80	0.90	0.5 (0.11 - 2.25)
309	anti-prothrombin IgG (>95 percentile)	0.80	1.80	2.25 (0.38 - 13.5)
30	APC ratio (T3 vs T1)	0.70	0.73	1.04 (0.42 - 2.59)
38	aPTT (T3 vs T1)	0.76	1.11	1.46 (0.59 - 3.62)
335	aPTT (for Protein C) (Q1 vs Q5)	1.53	2.43	1.59 (0.35 - 7.26)
39	bleeding time (T1 vs T3)	0.90	1.42	1.58 (0.19 - 12.93)
16	CLT (fibrinolytic potential) (>90 percentile)	2.60	1.90	0.73 (0.24 - 2.26)
300	CLT (hyperfibrinolysis vs. normofibrinolysis) (T3 vs T2)	2.82	1.50	0.53 (0.16 - 1.8)
314	CLT (hypofibrinolysis vs. normofibrinolysis) (T1 vs T2)	1.60	4.07	2.54 (0.71 - 9.09)
344	d-dimer (Q5 vs Q1)	1.49	1.09	0.73 (0.12 - 4.38)
351	d-dimer (Q4 vs Q1)	1.70	1.52	0.89 (0.39 - 2.06)
57	d-dimer (T3 vs T1)	1.59	1.62	1.02 (0.43 - 2.41)
21	d-dimer (T3 vs T1)	1.39	1.56	1.12 (0.52 - 2.42)
333	d-dimer (high vs low)	2.10	2.60	1.24 (0.27 - 5.63)
340	d-dimer (SD)	1.02	1.27	1.25 (0.72 - 2.16)
37	d-dimer (T3 vs T1)	1.45	2.09	1.44 (0.63 - 3.32)
64	d-dimer (SD (log scale))	1.04	1.96	1.88 (0.81 - 4.4)
28	F1+2 fragment (T3 vs T1)	1.03	0.96	0.93 (0.35 - 2.46)
58	F1+2 fragment (T3 vs T1)	0.92	1.00	1.09 (0.45 - 2.64)
1	fibrinogen (Q4 vs Q1)	2.18	1.26	0.58
106	fibrinogen (T1 vs T3)	0.62	0.40	0.65
342	fibrinogen (Q5 vs Q1)	2.45	1.63	0.67 (0.14 - 3.25)
293	fibrinogen (>300 mg/dl)	3.68	2.72	0.74 (0.11 - 5.05)
284	fibrinogen (SD)	1.30	1.01	0.78 (0.61 - 0.98)
348	fibrinogen (SD (log scale))	1.52	1.36	0.89 (0.65 - 1.24)
339	fibrinogen (SD)	1.13	1.14	1.01 (0.68 - 1.51)
69	fibrinogen (SD)	1.02	1.05	1.03 (0.69 - 1.53)
65	fibrinogen (Q4 vs Q1)	2.30	2.50	1.09 (0.61 - 1.93)
31	fibrinogen (T3 vs T1)	1.26	1.51	1.2 (0.56 - 2.55)
61	fibrinogen (T3 vs T1)	1.66	2.06	1.24 (0.56 - 2.75)
63	fibrinogen (SD (log scale))	1.04	1.52	1.46 (0.96 - 2.22)
107	fibrinogen (T1 vs T3)	0.56	0.94	1.68
53	fibrinopeptide A (T3 vs T1)	1.36	0.85	0.63 (0.31 - 1.26)
11	FII (Q4 vs Q1)	0.89	1.34	1.51 (0.32 - 7.04)
4	FIX (Q4 vs Q1)	0.93	0.96	1.03 (0.27 - 3.92)
6	FV (Q4 vs Q1)	1.22	1.59	1.3 (0.34 - 5.02)
55	FVII (T3 vs T1)	1.12	0.89	0.79 (0.31 - 2.05)
59	FVII (SD (log scale))	0.98	1.08	1.1 (0.63 - 1.93)
67	FVII:ag (SD)	1.11	1.01	0.91 (0.57 - 1.44)
68	FVII:c (SD)	1.01	0.93	0.92 (0.58 - 1.47)
3	FVII:c (Q4 vs Q1)	1.00	1.00	1.00
36	FVII:c (T3 vs T1)	0.65	0.91	1.4 (0.54 - 3.6)
349	FVII:c (unit log) (SD (log scale))	0.98	1.07	1.09 (0.74 - 1.6)
41	FVIII:c (SD)	1.20	1.16	0.97 (0.74 - 1.26)

42	FVIII:c (SD)	1.13	1.15	1.02 (0.78 - 1.32)
12	FVIII:c (Q4 vs Q1)	1.22	1.93	1.58
29	FVIII:c (T3 vs T1)	1.12	1.10	0.98 (0.4 - 2.41)
8	FX (Q4 vs Q1)	0.61	0.88	1.44 (0.37 - 5.66)
5	FXI (Q4 vs Q1)	1.27	1.62	1.28 (0.33 - 4.86)
290	FXIa-AT-INH (T3 vs T1)	1.31	1.22	0.93 (0.23 - 3.82)
310	FXIa-AT-INH (>90 percentile)	0.94	2.18	2.32 (0.68 - 7.95)
292	FXIa-C1-INH (T3 vs T1)	1.05	1.51	1.44 (0.41 - 5.02)
315	FXIa-C1-INH (>90 percentile)	1.13	2.92	2.58 (0.77 - 8.72)
2	FXII (Q4 vs Q1)	1.17	1.16	0.99 (0.27 - 3.7)
291	FXIIa-C1-INH (T3 vs T1)	0.73	0.86	1.18 (0.37 - 3.77)
313	FXIIa-C1-INH (>90 percentile)	0.74	1.87	2.53 (0.74 - 8.6)
289	KAL-C1-INH (T3 vs T1)	0.73	0.67	0.92 (0.28 - 2.99)
312	KAL-C1-INH (>90 percentile)	2.12	5.14	2.42 (0.77 - 7.64)
316	lupus anticoagulant (ratio >=1.15)	5.30	43.10	8.13 (0.61 - 108.76)
288	PAI-1 (Q4 vs Q1)	3.35	1.32	0.39 (0.08 - 1.88)
34	PAI-1 (T3 vs T1)	1.30	1.61	1.24 (0.49 - 3.13)
66	plasminogen (SD)	1.41	1.10	0.78 (0.42 - 1.44)
9	plasminogen (Q4 vs Q1)	0.81	1.20	1.48 (0.34 - 6.4)
25	PLT aggregation (first) (Q5 vs Q1)	1.31	0.64	0.49 (0.18 - 1.31)
33	PLT aggregation (irreversible) (high vs low)	1.04	1.26	1.21 (0.47 - 3.15)
35	PLT retention (Q5 vs Q1)	0.80	1.05	1.31 (0.39 - 4.43)
13	prot C (Q1 vs Q5)	0.92	1.52	1.65 (1.05 - 2.6)
10	prot C (high) (Q4 vs Q1)	1.03	1.54	1.50
26	TAT (T3 vs T1)	0.97	0.71	0.73 (0.27 - 1.95)
287	t-PA (Q4 vs Q1)	5.89	2.32	0.39 (0.07 - 2.07)
350	t-PA (Q4 vs Q1)	3.20	1.42	0.44 (0.17 - 1.15)
56	t-PA (T3 vs T1)	1.80	1.65	0.92 (0.38 - 2.22)
32	t-PA (T3 vs T1)	1.10	1.33	1.21 (0.54 - 2.73)
62	t-PA (SD (log scale))	1.25	1.69	1.35 (0.81 - 2.25)
22	t-PA (T3 vs T1)	0.92	1.38	1.5 (0.69 - 3.27)
341	t-PA (SD)	1.04	1.60	1.54 (0.55 - 4.34)
345	trombin generation (PEAK) (SD)	1.71	1.31	0.77 (0.38 - 1.53)
346	trombin generation (PEAK) (SD)	1.04	1.31	1.26 (0.76 - 2.1)
347	trombin generation (PEAK) (SD)	1.03	1.31	1.27 (0.83 - 1.95)
54	VWF (T3 vs T1)	1.53	1.02	0.67 (0.31 - 1.44)
343	VWF (Q5 vs Q1)	1.52	1.06	0.7 (0.16 - 3.06)
27	VWF (T3 vs T1)	1.09	0.97	0.89 (0.43 - 1.86)
332	VWF (Q4 vs Q1)	1.39	1.25	0.9 (0.41 - 1.97)
338	VWF (SD)	1.04	0.97	0.93 (0.65 - 1.34)
60	VWF (SD (log scale))	0.95	1.15	1.21 (0.76 - 1.92)
7	VWF (Q4 vs Q1)	1.21	1.71	1.41
23	VWF (T3 vs T1)	1.24	1.93	1.56 (0.72 - 3.34)
304	VWF (Q4 vs Q1)	4.20	6.70	1.6 (0.4 - 6.38)

ID, identification number; RR IS, relative risk for ischaemic stroke; RR MI, relative risk for myocardial infarction; RRR relative risk ratio.
