# Supplementary material for: Hypercoagulability Is a Stronger Risk Factor for Ischaemic Stroke than for Myocardial Infarction: A Systematic Review
Source: PLoS One. 2015 Aug 7;10(8):e0133523. doi: 10.1371/journal.pone.0133523 (PMC4529149; doi:10.1371/journal.pone.0133523)
Supplement: S3 Table — (PDF) [file pone.0133523.s006.pdf]

S3 Table. Genotypic measurements sorted alphabetically.

ID	Factor(contrast)	RR MI	RR IS	RRR (95% CI)
184	antithrombin SNP 1734 (allele)	1.02	1.01	0.99 (0.64 - 1.54)
244	antithrombin SNP 2415 (allele)	1.00	1.11	1.11 (0.75 - 1.65)
261	antithrombin SNP 5403 (allele)	0.91	1.06	1.16 (0.54 - 2.51)
173	antithrombin SNP 7199 (allele)	1.05	1.01	0.96 (0.62 - 1.49)
226	antithrombin SNP 9089 (allele)	1.01	1.08	1.07 (0.81 - 1.41)
260	FGA 251 (allele)	0.93	1.07	1.15 (0.87 - 1.52)
121	FGA 3807 (allele)	1.07	0.82	0.77 (0.52 - 1.12)
267	FGA 5498 (allele)	0.92	1.15	1.25 (0.84 - 1.86)
194	FGA 6534 (allele)	0.96	0.97	1.01 (0.77 - 1.33)
162	FGA 9205 (allele)	0.99	0.94	0.95 (0.66 - 1.37)
299	FGA SNP Thr312Ala rs6050 (allele)	0.82	0.43	0.52 (0.15 - 1.8)
212	FGB 1083 (allele)	0.97	1.01	1.04 (0.71 - 1.52)
177	FGB 11079 (allele)	1.02	0.99	0.97 (0.73 - 1.28)
139	FGB 1643 (allele)	1.02	0.91	0.89 (0.64 - 1.24)
134	FGB 9487 (allele)	1.00	0.87	0.87 (0.62 - 1.23)
306	FGB SNP 455G/A rs1800790 (allele)	0.98	1.76	1.8 (0.32 - 10.06)
183	FGG 129 (allele)	0.94	0.93	0.99 (0.73 - 1.34)
255	FGG 5836 (allele)	1.13	1.28	1.13 (0.57 - 2.24)
148	FGG 902 (allele)	1.10	1.02	0.93 (0.7 - 1.22)
234	FGG 9340 (allele)	0.94	1.02	1.09 (0.81 - 1.46)
296	FII SNP C148T (or G455A) (allele)	1.10	0.93	0.85 (0.45 - 1.6)
227	FII SNP 21239 (allele)	1.00	1.07	1.07 (0.82 - 1.4)
253	FII SNP 280 (allele)	0.94	1.06	1.13 (0.7 - 1.82)
259	FII SNP 3696 (allele)	1.03	1.18	1.15 (0.51 - 2.58)
252	FII SNP 4992 (allele)	1.03	1.16	1.13 (0.83 - 1.52)
257	FII SNP 5389 (allele)	1.01	1.15	1.14 (0.86 - 1.51)
215	FII SNP 5467 (allele)	1.09	1.14	1.05 (0.72 - 1.52)
214	FII SNP 7530 (allele)	1.10	1.15	1.05 (0.73 - 1.5)
319	FII SNP G20210A (dominant)	4.00	1.60	0.4 (0 - 77.58)
48	FII SNP G20210A (dominant)	1.70	1.10	0.65 (0.19 - 2.2)
285	FII SNP G20210A (dominant)	5.42	4.00	0.74 (0 - 112.99)
303	FII SNP G20210A (dominant)	1.00	1.00	1 (0.12 - 8.33)
242	FII SNP G20210A (allele)	1.39	1.54	1.11 (0.41 - 3.03)
297	FII SNP G20210A (dominant)	0.80	1.10	1.38 (0.31 - 6.05)
45	FII SNP G20210A (dominant)	0.88	1.40	1.59 (0.16 - 15.37)
152	FIX SNP 10948 (allele)	1.08	1.01	0.94 (0.4 - 2.2)
114	FIX SNP 12806 (allele)	1.54	0.99	0.64 (0.24 - 1.74)
250	FIX SNP 16171 (allele)	0.76	0.85	1.12 (0.44 - 2.84)
235	FIX SNP 21554 (allele)	0.99	1.08	1.09 (0.66 - 1.8)
185	FIX SNP 21975 (allele)	0.96	0.96	1 (0.6 - 1.66)
240	FIX SNP 27226 (allele)	1.04	1.15	1.11 (0.62 - 1.97)
124	FIX SNP 30893 (allele)	0.82	0.63	0.77 (0.28 - 2.08)
241	FIX SNP 35124 (allele)	1.04	1.15	1.11 (0.64 - 1.92)
136	FIX SNP 4135 (allele)	1.17	1.02	0.87 (0.55 - 1.39)
246	FIX SNP 6347 (allele)	0.94	1.05	1.12 (0.68 - 1.84)
223	FIX SNP 716 (allele)	1.00	1.06	1.06 (0.66 - 1.71)
318	FV Leiden (dominant)	2.40	0.00	#NULL!
46	FV Leiden (dominant)	0.81	0.42	0.52 (0.14 - 1.88)
47	FV Leiden (dominant)	0.80	0.51	0.64 (0.08 - 5)
295	FV Leiden (dominant)	1.50	1.00	0.67 (0.14 - 3.13)

49	FV Leiden (dominant)	0.83	0.68	0.82 (0.38 - 1.77)
50	FV Leiden (dominant)	0.91	0.88	0.97 (0.31 - 2.98)
51	FV Leiden (dominant)	0.85	0.96	1.13 (0.41 - 3.11)
286	FV Leiden (dominant)	3.75	4.28	1.14 (0.05 - 24.1)
334	FV Leiden (dominant)	0.77	1.12	1.45 (0.16 - 13.07)
305	FV Leiden (dominant)	1.10	1.80	1.64 (0.45 - 6)
105	FV Leiden (allele)	1.22	2.98	2.44 (0.6 - 10)
294	FV Leiden (dominant)	0.76	2.60	3.42 (0.11 - 104)
149	FV SNP 17557 (allele)	1.04	0.97	0.93 (0.7 - 1.25)
264	FV SNP lower 29565 (allele)	0.96	1.16	1.21 (0.87 - 1.69)
239	FV SNP lower 30539 (allele)	1.05	1.16	1.1 (0.72 - 1.7)
176	FV SNP lower 3578 (allele)	1.02	0.99	0.97 (0.74 - 1.27)
182	FV SNP lower 35788 (allele)	1.15	1.13	0.98 (0.58 - 1.65)
74	FV SNP Rs2269648 (allele)	1.11	0.86	0.77 (0.38 - 1.57)
87	FV SNP Rs2420369 (allele)	1.17	1.26	1.08 (0.51 - 2.28)
100	FV SNP Rs3753305 (allele)	0.98	1.36	1.39 (0.67 - 2.86)
96	FV SNP Rs6013 (allele)	1.12	1.41	1.26 (0.47 - 3.37)
82	FV SNP Rs6019 (allele)	1.13	1.13	1 (0.21 - 4.68)
97	FV SNP Rs6030 (allele)	1.11	1.41	1.27 (0.61 - 2.66)
102	FV SNP Rs6035 (allele)	1.26	2.19	1.74 (0.56 - 5.4)
104	FV SNP Rs7542281 (allele)	1.11	2.46	2.22 (0.65 - 7.56)
72	FV SNP Rs9332575 (allele)	1.05	0.68	0.65 (0.28 - 1.5)
84	FV SNP Rs9332590 (allele)	1.03	1.10	1.07 (0.55 - 2.08)
95	FV SNP Rs9332591 (allele)	1.14	1.39	1.22 (0.51 - 2.92)
90	FV SNP Rs9332618 (allele)	0.93	1.02	1.1 (0.51 - 2.38)
92	FV SNP Rs9332640 (allele)	1.22	1.40	1.15 (0.52 - 2.55)
76	FV SNP Rs9332695 (allele)	0.85	0.71	0.84 (0.24 - 2.85)
91	FV SNP Rs970741 (allele)	1.04	1.19	1.14 (0.55 - 2.4)
275	FV SNP upper 38592 (allele)	1.03	1.53	1.49 (0.69 - 3.21)
147	FV SNP upper 42713 (allele)	1.10	1.02	0.93 (0.7 - 1.23)
180	FV SNP upper 45765 (allele)	1.00	0.98	0.98 (0.56 - 1.71)
219	FV SNP upper 45888 (allele)	0.91	0.96	1.05 (0.78 - 1.42)
113	FV SNP upper 46058 (allele)	1.74	1.07	0.61 (0.21 - 1.83)
137	FV SNP upper 66464 (allele)	1.06	0.94	0.89 (0.51 - 1.53)
169	FV SNP upper 66872 (allele)	0.94	0.90	0.96 (0.68 - 1.35)
217	FV SNP upper 68717 (allele)	0.97	1.02	1.05 (0.8 - 1.37)
116	FV SNP upper 72877 (allele)	1.20	0.82	0.68 (0.35 - 1.32)
271	FVII SNP 115 (allele)	1.07	1.40	1.31 (0.28 - 6.14)
222	FVII SNP 15386 (allele)	1.00	1.06	1.06 (0.7 - 1.61)
112	FVII SNP 16826 (allele)	1.75	0.49	0.28 (0.03 - 2.4)
170	FVII SNP 18311 (allele)	0.97	0.93	0.96 (0.73 - 1.26)
198	FVII SNP 185 (allele)	0.95	0.97	1.02 (0.74 - 1.4)
192	FVII SNP 2643 (allele)	0.99	1.00	1.01 (0.76 - 1.34)
274	FVIII SNP 139972 (allele)	0.83	1.20	1.45 (0.68 - 3.08)
282	FVIII SNP 165293 rs6655259 (allele)	0.54	2.55	4.72 (0.62 - 35.73)
280	FVIII SNP 25167 (allele)	0.75	2.10	2.8 (0.7 - 11.2)
276	FVIII SNP 55941 (allele)	1.04	1.56	1.5 (0.9 - 2.5)
278	FVIII SNP 95826 (allele)	0.94	1.70	1.81 (1.02 - 3.2)
143	FVIII SNP 95910 (allele)	1.28	1.16	0.91 (0.42 - 1.96)
161	FX SNP 11962 (allele)	0.99	0.94	0.95 (0.73 - 1.24)
258	FX SNP 14881 (allele)	0.97	1.11	1.14 (0.79 - 1.65)
164	FX SNP 16893 (allele)	1.00	0.95	0.95 (0.74 - 1.22)
228	FX SNP 17396 (allele)	0.98	1.05	1.07 (0.8 - 1.44)

172	FX SNP 18352 (allele)	1.02	0.98	0.96 (0.64 - 1.45)
279	FX SNP 22739 (allele)	0.69	1.31	1.9 (0.21 - 16.96)
168	FX SNP 26242 (allele)	0.92	0.88	0.96 (0.66 - 1.38)
268	FX SNP 4544 (allele)	0.96	1.20	1.25 (0.83 - 1.87)
197	FX SNP 8946 (allele)	0.99	1.01	1.02 (0.78 - 1.33)
123	FX SNP 9501 (allele)	0.99	0.76	0.77 (0.51 - 1.17)
265	FXI SNP 10942 (allele)	1.06	1.29	1.22 (0.83 - 1.78)
202	FXI SNP 20423 (allele)	0.91	0.93	1.02 (0.72 - 1.46)
205	FXI SNP 228771 (allele)	1.00	1.03	1.03 (0.78 - 1.35)
165	FXI SNP 25455 (allele)	1.01	0.96	0.95 (0.71 - 1.26)
238	FXI SNP 26011 (allele)	1.06	1.17	1.1 (0.75 - 1.62)
128	FXI SNP 3450 (allele)	1.00	0.81	0.81 (0.59 - 1.11)
166	FXI SNP 3543 (allele)	1.04	0.99	0.95 (0.73 - 1.24)
277	FXI SNP 4197 (allele)	0.96	1.52	1.58 (0.61 - 4.1)
248	FXI SNP 6783 (allele)	1.10	1.23	1.12 (0.69 - 1.81)
337	FXII SNP (dominant)	4.80	4.10	0.85 (0.06 - 12.73)
122	FXII SNP 6570 (allele)	1.16	0.89	0.77 (0.34 - 1.75)
195	FXII SNP 7532 (allele)	1.04	1.06	1.02 (0.77 - 1.34)
247	FXIIIA SNP 148318 (allele)	1.02	1.14	1.12 (0.8 - 1.55)
140	FXIIIA SNP 165306 (allele)	1.12	1.01	0.9 (0.65 - 1.25)
157	FXIIIA SNP 165399 (allele)	1.04	0.98	0.94 (0.71 - 1.26)
144	FXIIIA SNP 170779 (allele)	1.12	1.02	0.91 (0.64 - 1.29)
167	FXIIIA SNP 176866 (allele)	1.08	1.03	0.95 (0.68 - 1.34)
281	FXIIIA SNP 177424 rs3024462 (allele)	0.49	1.82	3.71 (0.62 - 22.35)
178	FXIIIA SNP 177778 (allele)	1.12	1.09	0.97 (0.57 - 1.67)
269	FXIIIA SNP 4377 (allele)	0.92	1.16	1.26 (0.93 - 1.71)
115	FXIIIA SNP 72060 (allele)	1.00	0.67	0.67 (0.27 - 1.66)
301	FXIIIA SNP Pro564Leu (dominant)	1.40	0.89	0.64 (0.31 - 1.29)
324	FXIIIA SNP Pro564Leu (dominant)	0.80	0.99	1.24 (0.33 - 4.63)
328	FXIIIA SNP Tir204Phe (dominant)	1.02	1.95	1.91 (0.2 - 18.29)
317	FXIIIA SNP Tyr204phe (dominant)	0.82	9.10	11.1 (5.64 - 21.82)
302	FXIIIA SNP Val34Leu (dominant)	1.07	0.77	0.72 (0.36 - 1.43)
111	FXIIIA SNP Val34Leu (dominant)	1.00	1.33	1.33 (0.62 - 2.84)
325	FXIIIA SNP Val34Leu (Val/Leu vs Val/Val)	0.80	1.19	1.49 (0.4 - 5.5)
331	FXIIIA SNP Val34Leu (Leu/Leu vs Val/Val)	0.77	3.59	4.66 (0.44 - 49.1)
224	FXIIIB SNP 17686 (allele)	1.08	1.15	1.06 (0.78 - 1.45)
181	FXIIIB SNP 29759 (allele)	1.11	1.09	0.98 (0.75 - 1.28)
179	FXIIIB SNP 5995 (allele)	0.92	0.90	0.98 (0.75 - 1.28)
225	FXIIIB SNP 7319 (allele)	0.90	0.96	1.07 (0.69 - 1.66)
141	FXIIIB SNP 9706 (allele)	1.05	0.95	0.9 (0.64 - 1.29)
307	FXIIIB SNP His95Arg (dominant)	0.79	1.70	2.15 (0.88 - 5.25)
327	GPIa SNP C807T (recessive)	1.26	2.24	1.78 (0.45 - 7.04)
322	GPIa SNP glu/Lys (recessive)	1.06	0.96	0.91 (0.18 - 4.58)
330	GPIb SNP thr/Met (recessive)	0.58	1.48	2.55 (0.48 - 13.69)
109	GPIb-alpha SNP HPA-2 (allele)	2.09	2.40	1.15 (0.23 - 5.65)
110	GPIb-alpha VNTR (allele)	1.71	2.23	1.3 (0.31 - 5.43)
320	GPIIb SNP ile/Ser (recessive)	1.85	1.20	0.65 (0.18 - 2.37)
321	GPIIIa SNP Leu/pro (recessive)	1.14	1.01	0.89 (0.24 - 3.27)
88	ICAM1 SNP Rs281432 (allele)	1.22	1.32	1.08 (0.51 - 2.3)
70	ICAM1 SNP Rs3093030 (allele)	1.04	0.57	0.55 (0.26 - 1.17)
85	ICAM1 SNP Rs3093032 (allele)	1.08	1.16	1.07 (0.48 - 2.42)
94	ICAM1 SNP Rs5030341 (allele)	1.25	1.52	1.22 (0.57 - 2.59)
81	ICAM1 SNP Rs5030347 (allele)	0.97	0.97	1 (0.93 - 1.08)

98	ICAM1 SNP Rs5030390 (allele)	1.23	1.59	1.29 (0.3 - 5.61)
199	PAI-1 SNP 10381 (allele)	0.94	0.96	1.02 (0.63 - 1.66)
191	PAI-1 SNP 12219 (allele)	1.01	1.02	1.01 (0.79 - 1.3)
190	PAI-1 SNP 4588 (allele)	1.01	1.02	1.01 (0.68 - 1.49)
323	PAI-1 SNP 4G/5G (4G/4G vs 5G/5G)	0.40	0.49	1.23 (0.24 - 6.14)
326	PAI-1 SNP 4G/5G (allele)	0.50	0.84	1.68 (0.45 - 6.24)
329	PAI-1 SNP 4G/5G (4G/5G vs 4G/4G)	0.52	1.10	2.12 (0.51 - 8.69)
283	PAI-1 SNP 4G/5G promotor (4G/4G vs 5G/5G)	0.96	0.49	0.51 (0.11 - 2.4)
44	PAI-1 SNP 4G/5G promotor (allele)	0.93	1.10	1.18 (0.55 - 2.53)
218	PAI-1 SNP 5878 (allele)	0.95	1.00	1.05 (0.75 - 1.47)
245	PAI-1 SNP 664 (allele)	0.99	1.10	1.11 (0.84 - 1.48)
237	plasminogen SNP 1470 (allele)	1.00	1.10	1.1 (0.77 - 1.58)
251	plasminogen SNP 15255 (allele)	0.96	1.08	1.13 (0.87 - 1.46)
131	plasminogen SNP 18114 (allele)	1.21	1.01	0.83 (0.63 - 1.11)
155	plasminogen SNP 1983 (allele)	1.02	0.96	0.94 (0.67 - 1.33)
220	plasminogen SNP 2967 (allele)	1.04	1.10	1.06 (0.81 - 1.38)
203	plasminogen SNP 31439 (allele)	0.83	0.85	1.02 (0.75 - 1.39)
272	plasminogen SNP 34158 (allele)	1.20	1.58	1.32 (0.7 - 2.49)
146	plasminogen SNP 406 (allele)	0.97	0.89	0.92 (0.7 - 1.2)
236	plasminogen SNP 41108 (allele)	0.92	1.01	1.1 (0.77 - 1.57)
120	plasminogen SNP 41494 (allele)	1.15	0.87	0.76 (0.31 - 1.87)
153	plasminogen SNP 54925 (allele)	1.09	1.02	0.94 (0.51 - 1.72)
273	prot C receptor SNP 3600 (allele)	0.74	0.99	1.34 (0.6 - 2.97)
175	prot C receptor SNP 6196 (allele)	0.98	0.95	0.97 (0.73 - 1.28)
118	prot C receptor SNP 837 (allele)	1.00	0.74	0.74 (0.46 - 1.2)
196	prot C receptor SNP rs1415772 (allele)	1.02	1.04	1.02 (0.74 - 1.4)
208	prot C SNP 10454 (allele)	0.93	0.96	1.03 (0.74 - 1.44)
266	prot C SNP 11310 (allele)	0.95	1.16	1.22 (0.92 - 1.62)
216	prot C SNP 2583 (allele)	1.01	1.06	1.05 (0.8 - 1.37)
135	prot C SNP 3220 (allele)	1.00	0.87	0.87 (0.42 - 1.79)
130	prot C SNP 4515 (allele)	1.07	0.89	0.83 (0.63 - 1.1)
174	prot C SNP 4732 (allele)	0.97	0.94	0.97 (0.7 - 1.33)
133	prot C SNP 4919 (allele)	1.05	0.91	0.87 (0.67 - 1.13)
193	prot C SNP 5867 (allele)	0.98	0.99	1.01 (0.73 - 1.39)
101	prot C SNP Rs1401296 (allele)	1.04	1.48	1.42 (0.67 - 3.03)
78	prot C SNP Rs1799810 (allele)	1.13	1.08	0.96 (0.49 - 1.86)
103	prot C SNP Rs2069920 (allele)	0.79	1.52	1.92 (0.93 - 3.96)
80	prot C SNP Rs2069923 (allele)	1.11	1.09	0.98 (0.22 - 4.3)
73	prot C SNP Rs2069928 (allele)	0.96	0.73	0.76 (0.37 - 1.56)
77	prot C SNP Rs5937 (allele)	1.21	1.04	0.86 (0.44 - 1.66)
40	prot S rs 867186 (GG vs AA)	1.27	1.14	0.9 (0.24 - 3.4)
43	prot S rs2069948 (CC vs TT)	1.08	1.27	1.18 (0.8 - 1.74)
142	prot S SNP 13154 (allele)	1.06	0.96	0.91 (0.65 - 1.25)
233	prot S SNP 26890 (allele)	0.98	1.06	1.08 (0.7 - 1.67)
210	prot S SNP 288 (allele)	1.05	1.09	1.04 (0.74 - 1.45)
187	prot S SNP 430 (allele)	0.94	0.94	1 (0.76 - 1.31)
150	prot S SNP 66205 (allele)	1.07	1.00	0.93 (0.72 - 1.21)
206	prot S SNP 66847 (allele)	1.00	1.03	1.03 (0.66 - 1.62)
108	PSGL-1 VNTR (allele)	0.89	0.51	0.57 (0.25 - 1.33)
158	TAFI SNP 10152 (allele)	1.07	1.01	0.94 (0.7 - 1.28)
20	TAFI SNP 1040C/T (CC vs TT)	0.48	0.86	1.79 (0.45 - 7.11)
119	TAFI SNP 18857 (allele)	1.17	0.87	0.74 (0.35 - 1.59)
163	TAFI SNP 2103 (allele)	0.99	0.94	0.95 (0.65 - 1.39)

200	TAFI SNP 31427 (allele)	0.94	0.96	1.02 (0.77 - 1.35)
201	TAFI SNP 32627 (allele)	0.94	0.96	1.02 (0.77 - 1.35)
145	TAFI SNP 35605 (allele)	1.04	0.95	0.91 (0.69 - 1.21)
117	TAFI SNP 36326 (allele)	1.10	0.81	0.74 (0.37 - 1.47)
17	TAFI SNP -438G/A (GG vs AA)	0.91	0.96	1.05 (0.21 - 5.39)
230	TAFI SNP 47956 (allele)	1.05	1.13	1.08 (0.72 - 1.62)
188	TAFI SNP 48100 (allele)	0.87	0.87	1 (0.63 - 1.58)
132	TAFI SNP 4947 (allele)	1.30	1.11	0.85 (0.51 - 1.42)
15	TAFI SNP 505G/A (GG vs AA)	1.44	0.84	0.58 (0.14 - 2.41)
256	TAFI SNP 51208 (allele)	1.03	1.17	1.14 (0.86 - 1.5)
129	TAFI SNP 54691 (allele)	1.10	0.91	0.83 (0.61 - 1.12)
151	TAFI SNP 7826 (allele)	0.92	0.86	0.93 (0.72 - 1.22)
221	TF SNP 11185 (allele)	0.86	0.91	1.06 (0.52 - 2.16)
160	TF SNP 13925 (allele)	1.11	1.05	0.95 (0.71 - 1.27)
189	TF SNP 5334 (allele)	1.08	1.09	1.01 (0.74 - 1.37)
186	TF SNP 599 (allele)	0.92	0.92	1 (0.76 - 1.31)
213	TF SNP 7877 (allele)	1.12	1.17	1.04 (0.58 - 1.9)
159	TFPI SNP 1502 (allele)	1.10	1.04	0.95 (0.71 - 1.25)
138	TFPI SNP 21164 (allele)	1.09	0.97	0.89 (0.51 - 1.56)
262	TFPI SNP 2418 (allele)	0.90	1.05	1.17 (0.9 - 1.51)
243	TFPI SNP 34214 (allele)	0.91	1.01	1.11 (0.83 - 1.48)
209	TFPI SNP 3437 (allele)	1.08	1.12	1.04 (0.57 - 1.89)
207	thombomodulin SNP 5110 (allele)	0.96	0.99	1.03 (0.77 - 1.37)
232	thombomodulin SNP 4007 (allele)	1.02	1.10	1.08 (0.78 - 1.49)
231	thombomodulin SNP 5318 (allele)	1.03	1.11	1.08 (0.78 - 1.49)
127	thombomodulin SNP 6235 (allele)	1.09	0.88	0.81 (0.58 - 1.13)
83	thombomodulin SNP Rs1042580 (allele)	0.96	0.98	1.02 (0.49 - 2.14)
93	thombomodulin SNP Rs1962 (allele)	1.03	1.25	1.21 (0.56 - 2.64)
86	thombomodulin SNP Rs3176119 (allele)	0.79	0.85	1.08 (0.28 - 4.09)
71	thombomodulin SNP Rs3176123 (allele)	0.94	0.58	0.62 (0.3 - 1.28)
75	thombomodulin SNP Rs3216183 (allele)	1.02	0.81	0.79 (0.35 - 1.78)
99	thombomodulin SNP Rs6048519 (allele)	0.98	1.28	1.31 (0.63 - 2.69)
79	thombomodulin SNP Rs6082986 (allele)	1.00	0.98	0.98 (0.46 - 2.07)
89	thombomodulin SNP Rs6113909 (allele)	0.98	1.07	1.09 (0.48 - 2.49)
154	t-PA SNP 12047 (allele)	0.99	0.93	0.94 (0.73 - 1.21)
156	t-PA SNP 12264 (allele)	1.02	0.96	0.94 (0.6 - 1.47)
229	t-PA SNP 16039 (allele)	0.98	1.05	1.07 (0.82 - 1.41)
204	t-PA SNP 17825 (allele)	1.01	1.04	1.03 (0.79 - 1.35)
249	t-PA SNP 22323 (allele)	0.93	1.04	1.12 (0.79 - 1.59)
126	t-PA SNP 2586 (allele)	0.93	0.73	0.78 (0.41 - 1.51)
125	t-PA SNP 30619 (allele)	1.07	0.83	0.78 (0.51 - 1.18)
254	t-PA SNP 35171 (allele)	1.00	1.13	1.13 (0.85 - 1.51)
263	t-PA SNP 6388 (allele)	0.92	1.10	1.2 (0.74 - 1.92)
171	t-PA SNP 6971 (allele)	0.99	0.95	0.96 (0.6 - 1.53)
211	t-PA SNP 9823 (allele)	0.98	1.02	1.04 (0.8 - 1.36)
270	t-PA SNP 9944 (allele)	0.84	1.08	1.29 (0.66 - 2.5)
18	VWF SNP rs1063857 (dominant)	1.15	1.35	1.17 (0.64 - 2.16)
336	VWF SNP P475S (dominant)	0.56	0.98	1.75 (0.1 - 31.18)
19	VWF SNP rs216293 (dominant)	1.26	1.50	1.19 (0.69 - 2.05)
52	WWF SNP sma I (recessive)	1.81	3.29	1.82 (0.32 - 10.34)

ID, identification number; RR IS, relative risk for ischaemic stroke; RR MI, relative risk for myocardial infarction; RRR relative risk ratio.
