# Supplementary material for: Hypercoagulability Is a Stronger Risk Factor for Ischaemic Stroke than for Myocardial Infarction: A Systematic Review
Source: PLoS One. 2015 Aug 7;10(8):e0133523. doi: 10.1371/journal.pone.0133523 (PMC4529149; doi:10.1371/journal.pone.0133523)
Supplement: S4 Table — (PDF) [file pone.0133523.s007.pdf]

S4 Table. All prothrombotic factors sorted by ID.

ID	Factor(contrast)	RR MI	RR IS	RRR (95% CI)
1	fibrinogen (Q4 vs Q1)	2.18	1.26	0.58
2	FXII (Q4 vs Q1)	1.17	1.16	0.99 (0.27 - 3.7)
3	FVII:c (Q4 vs Q1)	1.00	1.00	1
4	FIX (Q4 vs Q1)	0.93	0.96	1.03 (0.27 - 3.92)
5	FXI (Q4 vs Q1)	1.27	1.62	1.28 (0.33 - 4.86)
6	FV (Q4 vs Q1)	1.22	1.59	1.3 (0.34 - 5.02)
7	VWF (Q4 vs Q1)	1.21	1.71	1.41
8	FX (Q4 vs Q1)	0.61	0.88	1.44 (0.37 - 5.66)
9	plasminogen (Q4 vs Q1)	0.81	1.20	1.48 (0.34 - 6.4)
10	prot C (high) (Q4 vs Q1)	1.03	1.54	1.50
11	FII (Q4 vs Q1)	0.89	1.34	1.51 (0.32 - 7.04)
12	FVIII:c (Q4 vs Q1)	1.22	1.93	1.58
13	prot C (Q1 vs Q5)	0.92	1.52	1.65 (1.05 - 2.6)
14	alpha2 antiplasmin (Q4 vs Q1)	0.81	1.36	1.68 (0.39 - 7.21)
15	TAFI SNP 505G/A (GG vs AA)	1.44	0.84	0.58 (0.14 - 2.41)
16	CLT (fibrinolytic potential) (>90 percentile)	2.60	1.90	0.73 (0.24 - 2.26)
17	TAFI SNP -438G/A (GG vs AA)	0.91	0.96	1.05 (0.21 - 5.39)
18	VWF SNP rs1063857 (dominant)	1.15	1.35	1.17 (0.64 - 2.16)
19	VWF SNP rs216293 (dominant)	1.26	1.50	1.19 (0.69 - 2.05)
20	TAFI SNP 1040C/T (CC vs TT)	0.48	0.86	1.79 (0.45 - 7.11)
21	d-dimer (T3 vs T1)	1.39	1.56	1.12 (0.52 - 2.42)
22	t-PA (T3 vs T1)	0.92	1.38	1.5 (0.69 - 3.27)
23	VWF (T3 vs T1)	1.24	1.93	1.56 (0.72 - 3.34)
24	aggregation (whole blood) (Q5 vs Q1)	1.00	0.25	0.25 (0.07 - 0.93)
25	PLT aggregation (first) (Q5 vs Q1)	1.31	0.64	0.49 (0.18 - 1.31)
26	TAT (T3 vs T1)	0.97	0.71	0.73 (0.27 - 1.95)
27	VWF (T3 vs T1)	1.09	0.97	0.89 (0.43 - 1.86)
28	F1+2 fragment (T3 vs T1)	1.03	0.96	0.93 (0.35 - 2.46)
29	FVIII:c (T3 vs T1)	1.12	1.10	0.98 (0.4 - 2.41)
30	APC ratio (T3 vs T1)	0.70	0.73	1.04 (0.42 - 2.59)
31	fibrinogen (T3 vs T1)	1.26	1.51	1.2 (0.56 - 2.55)
32	t-PA (T3 vs T1)	1.10	1.33	1.21 (0.54 - 2.73)
33	PLT aggregation (irreversible) (high vs low)	1.04	1.26	1.21 (0.47 - 3.15)
34	PAI-1 (T3 vs T1)	1.30	1.61	1.24 (0.49 - 3.13)
35	PLT retention (Q5 vs Q1)	0.80	1.05	1.31 (0.39 - 4.43)
36	FVII:c (T3 vs T1)	0.65	0.91	1.4 (0.54 - 3.6)
37	d-dimer (T3 vs T1)	1.45	2.09	1.44 (0.63 - 3.32)
38	aPTT (T3 vs T1)	0.76	1.11	1.46 (0.59 - 3.62)
39	bleeding time (T1 vs T3)	0.90	1.42	1.58 (0.19 - 12.93)
40	prot S rs 867186 (GG vs AA)	1.27	1.14	0.9 (0.24 - 3.4)
41	FVIII:c (SD)	1.20	1.16	0.97 (0.74 - 1.26)
42	FVIII:c (SD)	1.13	1.15	1.02 (0.78 - 1.32)
43	prot S rs2069948 (CC vs TT)	1.08	1.27	1.18 (0.8 - 1.74)
44	PAI-1 SNP 4G/5G promotor (allele)	0.93	1.10	1.18 (0.55 - 2.53)
45	FII SNP G20210A (dominant)	0.88	1.40	1.59 (0.16 - 15.37)
46	FV Leiden (dominant)	0.81	0.42	0.52 (0.14 - 1.88)
47	FV Leiden (dominant)	0.80	0.51	0.64 (0.08 - 5)
48	FII SNP G20210A (dominant)	1.70	1.10	0.65 (0.19 - 2.2)
49	FV Leiden (dominant)	0.83	0.68	0.82 (0.38 - 1.77)
50	FV Leiden (dominant)	0.91	0.88	0.97 (0.31 - 2.98)

51	FV Leiden (dominant)	0.85	0.96	1.13 (0.41 - 3.11)
52	WWF SNP sma I (recessive)	1.81	3.29	1.82 (0.32 - 10.34)
53	fibrinopeptide A (T3 vs T1)	1.36	0.85	0.63 (0.31 - 1.26)
54	VWF (T3 vs T1)	1.53	1.02	0.67 (0.31 - 1.44)
55	FVII (T3 vs T1)	1.12	0.89	0.79 (0.31 - 2.05)
56	t-PA (T3 vs T1)	1.80	1.65	0.92 (0.38 - 2.22)
57	d-dimer (T3 vs T1)	1.59	1.62	1.02 (0.43 - 2.41)
58	F1+2 fragment (T3 vs T1)	0.92	1.00	1.09 (0.45 - 2.64)
59	FVII (SD (log scale))	0.98	1.08	1.1 (0.63 - 1.93)
60	VWF (SD (log scale))	0.95	1.15	1.21 (0.76 - 1.92)
61	fibrinogen (T3 vs T1)	1.66	2.06	1.24 (0.56 - 2.75)
62	t-PA (SD (log scale))	1.25	1.69	1.35 (0.81 - 2.25)
63	fibrinogen (SD (log scale))	1.04	1.52	1.46 (0.96 - 2.22)
64	d-dimer (SD (log scale))	1.04	1.96	1.88 (0.81 - 4.4)
65	fibrinogen (Q4 vs Q1)	2.30	2.50	1.09 (0.61 - 1.93)
66	plasminogen (SD)	1.41	1.10	0.78 (0.42 - 1.44)
67	FVII:ag (SD)	1.11	1.01	0.91 (0.57 - 1.44)
68	FVII:c (SD)	1.01	0.93	0.92 (0.58 - 1.47)
69	fibrinogen (SD)	1.02	1.05	1.03 (0.69 - 1.53)
70	ICAM1 SNP Rs3093030 (allele)	1.04	0.57	0.55 (0.26 - 1.17)
71	thombomodulin SNP Rs3176123 (allele)	0.94	0.58	0.62 (0.3 - 1.28)
72	FV SNP Rs9332575 (allele)	1.05	0.68	0.65 (0.28 - 1.5)
73	prot C SNP Rs2069928 (allele)	0.96	0.73	0.76 (0.37 - 1.56)
74	FV SNP Rs2269648 (allele)	1.11	0.86	0.77 (0.38 - 1.57)
75	thombomodulin SNP Rs3216183 (allele)	1.02	0.81	0.79 (0.35 - 1.78)
76	FV SNP Rs9332695 (allele)	0.85	0.71	0.84 (0.24 - 2.85)
77	prot C SNP Rs5937 (allele)	1.21	1.04	0.86 (0.44 - 1.66)
78	prot C SNP Rs1799810 (allele)	1.13	1.08	0.96 (0.49 - 1.86)
79	thombomodulin SNP Rs6082986 (allele)	1.00	0.98	0.98 (0.46 - 2.07)
80	prot C SNP Rs2069923 (allele)	1.11	1.09	0.98 (0.22 - 4.3)
81	ICAM1 SNP Rs5030347 (allele)	0.97	0.97	1 (0.93 - 1.08)
82	FV SNP Rs6019 (allele)	1.13	1.13	1 (0.21 - 4.68)
83	thombomodulin SNP Rs1042580 (allele)	0.96	0.98	1.02 (0.49 - 2.14)
84	FV SNP Rs9332590 (allele)	1.03	1.10	1.07 (0.55 - 2.08)
85	ICAM1 SNP Rs3093032 (allele)	1.08	1.16	1.07 (0.48 - 2.42)
86	thombomodulin SNP Rs3176119 (allele)	0.79	0.85	1.08 (0.28 - 4.09)
87	FV SNP Rs2420369 (allele)	1.17	1.26	1.08 (0.51 - 2.28)
88	ICAM1 SNP Rs281432 (allele)	1.22	1.32	1.08 (0.51 - 2.3)
89	thombomodulin SNP Rs6113909 (allele)	0.98	1.07	1.09 (0.48 - 2.49)
90	FV SNP Rs9332618 (allele)	0.93	1.02	1.1 (0.51 - 2.38)
91	FV SNP Rs970741 (allele)	1.04	1.19	1.14 (0.55 - 2.4)
92	FV SNP Rs9332640 (allele)	1.22	1.40	1.15 (0.52 - 2.55)
93	thombomodulin SNP Rs1962 (allele)	1.03	1.25	1.21 (0.56 - 2.64)
94	ICAM1 SNP Rs5030341 (allele)	1.25	1.52	1.22 (0.57 - 2.59)
95	FV SNP Rs9332591 (allele)	1.14	1.39	1.22 (0.51 - 2.92)
96	FV SNP Rs6013 (allele)	1.12	1.41	1.26 (0.47 - 3.37)
97	FV SNP Rs6030 (allele)	1.11	1.41	1.27 (0.61 - 2.66)
98	ICAM1 SNP Rs5030390 (allele)	1.23	1.59	1.29 (0.3 - 5.61)
99	thombomodulin SNP Rs6048519 (allele)	0.98	1.28	1.31 (0.63 - 2.69)
100	FV SNP Rs3753305 (allele)	0.98	1.36	1.39 (0.67 - 2.86)
101	prot C SNP Rs1401296 (allele)	1.04	1.48	1.42 (0.67 - 3.03)
102	FV SNP Rs6035 (allele)	1.26	2.19	1.74 (0.56 - 5.4)
103	prot C SNP Rs2069920 (allele)	0.79	1.52	1.92 (0.93 - 3.96)

104	FV SNP Rs7542281 (allele)	1.11	2.46	2.22 (0.65 - 7.56)
105	FV Leiden (allele)	1.22	2.98	2.44 (0.6 - 10)
106	fibrinogen (T1 vs T3)	0.62	0.40	0.65
107	fibrinogen (T1 vs T3)	0.56	0.94	1.68
108	PSGL-1 VNTR (allele)	0.89	0.51	0.57 (0.25 - 1.33)
109	GPIb-alpha SNP HPA-2 (allele)	2.09	2.40	1.15 (0.23 - 5.65)
110	GPIb-alpha VNTR (allele)	1.71	2.23	1.3 (0.31 - 5.43)
111	FXIIIA SNP Val34Leu (dominant)	1.00	1.33	1.33 (0.62 - 2.84)
112	FVII SNP 16826 (allele)	1.75	0.49	0.28 (0.03 - 2.4)
113	FV SNP upper 46058 (allele)	1.74	1.07	0.61 (0.21 - 1.83)
114	FIX SNP 12806 (allele)	1.54	0.99	0.64 (0.24 - 1.74)
115	FXIIIA SNP 72060 (allele)	1.00	0.67	0.67 (0.27 - 1.66)
116	FV SNP upper 72877 (allele)	1.20	0.82	0.68 (0.35 - 1.32)
117	TAFI SNP 36326 (allele)	1.10	0.81	0.74 (0.37 - 1.47)
118	prot C receptor SNP 837 (allele)	1.00	0.74	0.74 (0.46 - 1.2)
119	TAFI SNP 18857 (allele)	1.17	0.87	0.74 (0.35 - 1.59)
120	plasminogen SNP 41494 (allele)	1.15	0.87	0.76 (0.31 - 1.87)
121	FGA 3807 (allele)	1.07	0.82	0.77 (0.52 - 1.12)
122	FXII SNP 6570 (allele)	1.16	0.89	0.77 (0.34 - 1.75)
123	FX SNP 9501 (allele)	0.99	0.76	0.77 (0.51 - 1.17)
124	FIX SNP 30893 (allele)	0.82	0.63	0.77 (0.28 - 2.08)
125	t-PA SNP 30619 (allele)	1.07	0.83	0.78 (0.51 - 1.18)
126	t-PA SNP 2586 (allele)	0.93	0.73	0.78 (0.41 - 1.51)
127	thombomodulin SNP 6235 (allele)	1.09	0.88	0.81 (0.58 - 1.13)
128	FXI SNP 3450 (allele)	1.00	0.81	0.81 (0.59 - 1.11)
129	TAFI SNP 54691 (allele)	1.10	0.91	0.83 (0.61 - 1.12)
130	prot C SNP 4515 (allele)	1.07	0.89	0.83 (0.63 - 1.1)
131	plasminogen SNP 18114 (allele)	1.21	1.01	0.83 (0.63 - 1.11)
132	TAFI SNP 4947 (allele)	1.30	1.11	0.85 (0.51 - 1.42)
133	prot C SNP 4919 (allele)	1.05	0.91	0.87 (0.67 - 1.13)
134	FGB 9487 (allele)	1.00	0.87	0.87 (0.62 - 1.23)
135	prot C SNP 3220 (allele)	1.00	0.87	0.87 (0.42 - 1.79)
136	FIX SNP 4135 (allele)	1.17	1.02	0.87 (0.55 - 1.39)
137	FV SNP upper 66464 (allele)	1.06	0.94	0.89 (0.51 - 1.53)
138	TFPI SNP 21164 (allele)	1.09	0.97	0.89 (0.51 - 1.56)
139	FGB 1643 (allele)	1.02	0.91	0.89 (0.64 - 1.24)
140	FXIIIA SNP 165306 (allele)	1.12	1.01	0.9 (0.65 - 1.25)
141	FXIIIB SNP 9706 (allele)	1.05	0.95	0.9 (0.64 - 1.29)
142	prot S SNP 13154 (allele)	1.06	0.96	0.91 (0.65 - 1.25)
143	FVIII SNP 95910 (allele)	1.28	1.16	0.91 (0.42 - 1.96)
144	FXIIIA SNP 170779 (allele)	1.12	1.02	0.91 (0.64 - 1.29)
145	TAFI SNP 35605 (allele)	1.04	0.95	0.91 (0.69 - 1.21)
146	plasminogen SNP 406 (allele)	0.97	0.89	0.92 (0.7 - 1.2)
147	FV SNP upper 42713 (allele)	1.10	1.02	0.93 (0.7 - 1.23)
148	FGG 902 (allele)	1.10	1.02	0.93 (0.7 - 1.22)
149	FV SNP 17557 (allele)	1.04	0.97	0.93 (0.7 - 1.25)
150	prot S SNP 66205 (allele)	1.07	1.00	0.93 (0.72 - 1.21)
151	TAFI SNP 7826 (allele)	0.92	0.86	0.93 (0.72 - 1.22)
152	FIX SNP 10948 (allele)	1.08	1.01	0.94 (0.4 - 2.2)
153	plasminogen SNP 54925 (allele)	1.09	1.02	0.94 (0.51 - 1.72)
154	t-PA SNP 12047 (allele)	0.99	0.93	0.94 (0.73 - 1.21)
155	plasminogen SNP 1983 (allele)	1.02	0.96	0.94 (0.67 - 1.33)
156	t-PA SNP 12264 (allele)	1.02	0.96	0.94 (0.6 - 1.47)

157	FXIIIA SNP 165399 (allele)	1.04	0.98	0.94 (0.71 - 1.26)
158	TAFI SNP 10152 (allele)	1.07	1.01	0.94 (0.7 - 1.28)
159	TFPI SNP 1502 (allele)	1.10	1.04	0.95 (0.71 - 1.25)
160	TF SNP 13925 (allele)	1.11	1.05	0.95 (0.71 - 1.27)
161	FX SNP 11962 (allele)	0.99	0.94	0.95 (0.73 - 1.24)
162	FGA 9205 (allele)	0.99	0.94	0.95 (0.66 - 1.37)
163	TAFI SNP 2103 (allele)	0.99	0.94	0.95 (0.65 - 1.39)
164	FX SNP 16893 (allele)	1.00	0.95	0.95 (0.74 - 1.22)
165	FXI SNP 25455 (allele)	1.01	0.96	0.95 (0.71 - 1.26)
166	FXI SNP 3543 (allele)	1.04	0.99	0.95 (0.73 - 1.24)
167	FXIIIA SNP 176866 (allele)	1.08	1.03	0.95 (0.68 - 1.34)
168	FX SNP 26242 (allele)	0.92	0.88	0.96 (0.66 - 1.38)
169	FV SNP upper 66872 (allele)	0.94	0.90	0.96 (0.68 - 1.35)
170	FVII SNP 18311 (allele)	0.97	0.93	0.96 (0.73 - 1.26)
171	t-PA SNP 6971 (allele)	0.99	0.95	0.96 (0.6 - 1.53)
172	FX SNP 18352 (allele)	1.02	0.98	0.96 (0.64 - 1.45)
173	antithrombin SNP 7199 (allele)	1.05	1.01	0.96 (0.62 - 1.49)
174	prot C SNP 4732 (allele)	0.97	0.94	0.97 (0.7 - 1.33)
175	prot C receptor SNP 6196 (allele)	0.98	0.95	0.97 (0.73 - 1.28)
176	FV SNP lower 3578 (allele)	1.02	0.99	0.97 (0.74 - 1.27)
177	FGB 11079 (allele)	1.02	0.99	0.97 (0.73 - 1.28)
178	FXIIIA SNP 177778 (allele)	1.12	1.09	0.97 (0.57 - 1.67)
179	FXIIIB SNP 5995 (allele)	0.92	0.90	0.98 (0.75 - 1.28)
180	FV SNP upper 45765 (allele)	1.00	0.98	0.98 (0.56 - 1.71)
181	FXIIIB SNP 29759 (allele)	1.11	1.09	0.98 (0.75 - 1.28)
182	FV SNP lower 35788 (allele)	1.15	1.13	0.98 (0.58 - 1.65)
183	FGG 129 (allele)	0.94	0.93	0.99 (0.73 - 1.34)
184	antithrombin SNP 1734 (allele)	1.02	1.01	0.99 (0.64 - 1.54)
185	FIX SNP 21975 (allele)	0.96	0.96	1 (0.6 - 1.66)
186	TF SNP 599 (allele)	0.92	0.92	1 (0.76 - 1.31)
187	prot S SNP 430 (allele)	0.94	0.94	1 (0.76 - 1.31)
188	TAFI SNP 48100 (allele)	0.87	0.87	1 (0.63 - 1.58)
189	TF SNP 5334 (allele)	1.08	1.09	1.01 (0.74 - 1.37)
190	PAI-1 SNP 4588 (allele)	1.01	1.02	1.01 (0.68 - 1.49)
191	PAI-1 SNP 12219 (allele)	1.01	1.02	1.01 (0.79 - 1.3)
192	FVII SNP 2643 (allele)	0.99	1.00	1.01 (0.76 - 1.34)
193	prot C SNP 5867 (allele)	0.98	0.99	1.01 (0.73 - 1.39)
194	FGA 6534 (allele)	0.96	0.97	1.01 (0.77 - 1.33)
195	FXII SNP 7532 (allele)	1.04	1.06	1.02 (0.77 - 1.34)
196	prot C receptor SNP rs1415772 (allele)	1.02	1.04	1.02 (0.74 - 1.4)
197	FX SNP 8946 (allele)	0.99	1.01	1.02 (0.78 - 1.33)
198	FVII SNP 185 (allele)	0.95	0.97	1.02 (0.74 - 1.4)
199	PAI-1 SNP 10381 (allele)	0.94	0.96	1.02 (0.63 - 1.66)
200	TAFI SNP 31427 (allele)	0.94	0.96	1.02 (0.77 - 1.35)
201	TAFI SNP 32627 (allele)	0.94	0.96	1.02 (0.77 - 1.35)
202	FXI SNP 20423 (allele)	0.91	0.93	1.02 (0.72 - 1.46)
203	plasminogen SNP 31439 (allele)	0.83	0.85	1.02 (0.75 - 1.39)
204	t-PA SNP 17825 (allele)	1.01	1.04	1.03 (0.79 - 1.35)
205	FXI SNP 228771 (allele)	1.00	1.03	1.03 (0.78 - 1.35)
206	prot S SNP 66847 (allele)	1.00	1.03	1.03 (0.66 - 1.62)
207	thombomodulin SNP 5110 (allele)	0.96	0.99	1.03 (0.77 - 1.37)
208	prot C SNP 10454 (allele)	0.93	0.96	1.03 (0.74 - 1.44)
209	TFPI SNP 3437 (allele)	1.08	1.12	1.04 (0.57 - 1.89)

210	prot S SNP 288 (allele)	1.05	1.09	1.04 (0.74 - 1.45)
211	t-PA SNP 9823 (allele)	0.98	1.02	1.04 (0.8 - 1.36)
212	FGB 1083 (allele)	0.97	1.01	1.04 (0.71 - 1.52)
213	TF SNP 7877 (allele)	1.12	1.17	1.04 (0.58 - 1.9)
214	FII SNP 7530 (allele)	1.10	1.15	1.05 (0.73 - 1.5)
215	FII SNP 5467 (allele)	1.09	1.14	1.05 (0.72 - 1.52)
216	prot C SNP 2583 (allele)	1.01	1.06	1.05 (0.8 - 1.37)
217	FV SNP upper 68717 (allele)	0.97	1.02	1.05 (0.8 - 1.37)
218	PAI-1 SNP 5878 (allele)	0.95	1.00	1.05 (0.75 - 1.47)
219	FV SNP upper 45888 (allele)	0.91	0.96	1.05 (0.78 - 1.42)
220	plasminogen SNP 2967 (allele)	1.04	1.10	1.06 (0.81 - 1.38)
221	TF SNP 11185 (allele)	0.86	0.91	1.06 (0.52 - 2.16)
222	FVII SNP 15386 (allele)	1.00	1.06	1.06 (0.7 - 1.61)
223	FIX SNP 716 (allele)	1.00	1.06	1.06 (0.66 - 1.71)
224	FXIIIB SNP 17686 (allele)	1.08	1.15	1.06 (0.78 - 1.45)
225	FXIIIB SNP 7319 (allele)	0.90	0.96	1.07 (0.69 - 1.66)
226	antithrombin SNP 9089 (allele)	1.01	1.08	1.07 (0.81 - 1.41)
227	FII SNP 21239 (allele)	1.00	1.07	1.07 (0.82 - 1.4)
228	FX SNP 17396 (allele)	0.98	1.05	1.07 (0.8 - 1.44)
229	t-PA SNP 16039 (allele)	0.98	1.05	1.07 (0.82 - 1.41)
230	TAFI SNP 47956 (allele)	1.05	1.13	1.08 (0.72 - 1.62)
231	thombomodulin SNP 5318 (allele)	1.03	1.11	1.08 (0.78 - 1.49)
232	thombomodulin SNP 4007 (allele)	1.02	1.10	1.08 (0.78 - 1.49)
233	prot S SNP 26890 (allele)	0.98	1.06	1.08 (0.7 - 1.67)
234	FGG 9340 (allele)	0.94	1.02	1.09 (0.81 - 1.46)
235	FIX SNP 21554 (allele)	0.99	1.08	1.09 (0.66 - 1.8)
236	plasminogen SNP 41108 (allele)	0.92	1.01	1.1 (0.77 - 1.57)
237	plasminogen SNP 1470 (allele)	1.00	1.10	1.1 (0.77 - 1.58)
238	FXI SNP 26011 (allele)	1.06	1.17	1.1 (0.75 - 1.62)
239	FV SNP lower 30539 (allele)	1.05	1.16	1.1 (0.72 - 1.7)
240	FIX SNP 27226 (allele)	1.04	1.15	1.11 (0.62 - 1.97)
241	FIX SNP 35124 (allele)	1.04	1.15	1.11 (0.64 - 1.92)
242	FII SNP G20210A (allele)	1.39	1.54	1.11 (0.41 - 3.03)
243	TFPI SNP 34214 (allele)	0.91	1.01	1.11 (0.83 - 1.48)
244	antithrombin SNP 2415 (allele)	1.00	1.11	1.11 (0.75 - 1.65)
245	PAI-1 SNP 664 (allele)	0.99	1.10	1.11 (0.84 - 1.48)
246	FIX SNP 6347 (allele)	0.94	1.05	1.12 (0.68 - 1.84)
247	FXIIIA SNP 148318 (allele)	1.02	1.14	1.12 (0.8 - 1.55)
248	FXI SNP 6783 (allele)	1.10	1.23	1.12 (0.69 - 1.81)
249	t-PA SNP 22323 (allele)	0.93	1.04	1.12 (0.79 - 1.59)
250	FIX SNP 16171 (allele)	0.76	0.85	1.12 (0.44 - 2.84)
251	plasminogen SNP 15255 (allele)	0.96	1.08	1.13 (0.87 - 1.46)
252	FII SNP 4992 (allele)	1.03	1.16	1.13 (0.83 - 1.52)
253	FII SNP 280 (allele)	0.94	1.06	1.13 (0.7 - 1.82)
254	t-PA SNP 35171 (allele)	1.00	1.13	1.13 (0.85 - 1.51)
255	FGG 5836 (allele)	1.13	1.28	1.13 (0.57 - 2.24)
256	TAFI SNP 51208 (allele)	1.03	1.17	1.14 (0.86 - 1.5)
257	FII SNP 5389 (allele)	1.01	1.15	1.14 (0.86 - 1.51)
258	FX SNP 14881 (allele)	0.97	1.11	1.14 (0.79 - 1.65)
259	FII SNP 3696 (allele)	1.03	1.18	1.15 (0.51 - 2.58)
260	FGA 251 (allele)	0.93	1.07	1.15 (0.87 - 1.52)
261	antithrombin SNP 5403 (allele)	0.91	1.06	1.16 (0.54 - 2.51)
262	TFPI SNP 2418 (allele)	0.90	1.05	1.17 (0.9 - 1.51)

263	t-PA SNP 6388 (allele)	0.92	1.10	1.2 (0.74 - 1.92)
264	FV SNP lower 29565 (allele)	0.96	1.16	1.21 (0.87 - 1.69)
265	FXI SNP 10942 (allele)	1.06	1.29	1.22 (0.83 - 1.78)
266	prot C SNP 11310 (allele)	0.95	1.16	1.22 (0.92 - 1.62)
267	FGA 5498 (allele)	0.92	1.15	1.25 (0.84 - 1.86)
268	FX SNP 4544 (allele)	0.96	1.20	1.25 (0.83 - 1.87)
269	FXIIIA SNP 4377 (allele)	0.92	1.16	1.26 (0.93 - 1.71)
270	t-PA SNP 9944 (allele)	0.84	1.08	1.29 (0.66 - 2.5)
271	FVII SNP 115 (allele)	1.07	1.40	1.31 (0.28 - 6.14)
272	plasminogen SNP 34158 (allele)	1.20	1.58	1.32 (0.7 - 2.49)
273	prot C receptor SNP 3600 (allele)	0.74	0.99	1.34 (0.6 - 2.97)
274	FVIII SNP 139972 (allele)	0.83	1.20	1.45 (0.68 - 3.08)
275	FV SNP upper 38592 (allele)	1.03	1.53	1.49 (0.69 - 3.21)
276	FVIII SNP 55941 (allele)	1.04	1.56	1.5 (0.9 - 2.5)
277	FXI SNP 4197 (allele)	0.96	1.52	1.58 (0.61 - 4.1)
278	FVIII SNP 95826 (allele)	0.94	1.70	1.81 (1.02 - 3.2)
279	FX SNP 22739 (allele)	0.69	1.31	1.9 (0.21 - 16.96)
280	FVIII SNP 25167 (allele)	0.75	2.10	2.8 (0.7 - 11.2)
281	FXIIIA SNP 177424 rs3024462 (allele)	0.49	1.82	3.71 (0.62 - 22.35)
282	FVIII SNP 165293 rs6655259 (allele)	0.54	2.55	4.72 (0.62 - 35.73)
283	PAI-1 SNP 4G/5G promotor (4G/4G vs 5G/5G)	0.96	0.49	0.51 (0.11 - 2.4)
284	fibrinogen (SD)	1.30	1.01	0.78 (0.61 - 0.98)
285	FII SNP G20210A (dominant)	5.42	4.00	0.74 (0 - 112.99)
286	FV Leiden (dominant)	3.75	4.28	1.14 (0.05 - 24.1)
287	t-PA (Q4 vs Q1)	5.89	2.32	0.39 (0.07 - 2.07)
288	PAI-1 (Q4 vs Q1)	3.35	1.32	0.39 (0.08 - 1.88)
289	KAL-C1-INH (T3 vs T1)	0.73	0.67	0.92 (0.28 - 2.99)
290	FXIa-AT-INH (T3 vs T1)	1.31	1.22	0.93 (0.23 - 3.82)
291	FXIIa-C1-INH (T3 vs T1)	0.73	0.86	1.18 (0.37 - 3.77)
292	FXIa-C1-INH (T3 vs T1)	1.05	1.51	1.44 (0.41 - 5.02)
293	fibrinogen (>300 mg/dl)	3.68	2.72	0.74 (0.11 - 5.05)
294	FV Leiden (dominant)	0.76	2.60	3.42 (0.11 - 104)
295	FV Leiden (dominant)	1.50	1.00	0.67 (0.14 - 3.13)
296	fibrinogen SNP C148T (or G455A) (allele)	1.10	0.93	0.85 (0.45 - 1.6)
297	FII SNP G20210A (dominant)	0.80	1.10	1.38 (0.31 - 6.05)
298	anti-cardiolipin IgG (>95 percentile)	1.80	0.90	0.5 (0.11 - 2.25)
299	FGA SNP Thr312Ala rs6050 (allele)	0.82	0.43	0.52 (0.15 - 1.8)
300	CLT (hyperfibrinolysis vs normofibrinolysis) (T3 vs T2)	2.82	1.50	0.53 (0.16 - 1.8)
301	FXIIIA SNP Pro564Leu (dominant)	1.40	0.89	0.64 (0.31 - 1.29)
302	FXIIIA SNP Val34Leu (dominant)	1.07	0.77	0.72 (0.36 - 1.43)
303	FII SNP G20210A (dominant)	1.00	1.00	1 (0.12 - 8.33)
304	VWF (Q4 vs Q1)	4.20	6.70	1.6 (0.4 - 6.38)
305	FV Leiden (dominant)	1.10	1.80	1.64 (0.45 - 6)
306	FGB SNP 455G/A rs1800790 (allele)	0.98	1.76	1.8 (0.32 - 10.06)
307	FXIIIB SNP His95Arg (dominant)	0.79	1.70	2.15 (0.88 - 5.25)
308	ADAMTS13 (Q1 vs Q4)	1.40	3.10	2.21 (0.65 - 7.51)
309	anti-prothrombin IgG (>95 percentile)	0.80	1.80	2.25 (0.38 - 13.5)
310	FXIa-AT-INH (>90 percentile)	0.94	2.18	2.32 (0.68 - 7.95)
311	anti-beta2GP (>95 percentile)	1.20	2.80	2.33 (0.63 - 8.71)
312	KAL-C1-INH (>90 percentile)	2.12	5.14	2.42 (0.77 - 7.64)
313	FXIIa-C1-INH (>90 percentile)	0.74	1.87	2.53 (0.74 - 8.6)

314	CLT (hypofibrinolysis vs. normofibrinolysis) (T1 vs T2)	1.60	4.07	2.54 (0.71 - 9.09)
315	FXIa-C1-INH (>90 percentile)	1.13	2.92	2.58 (0.77 - 8.72)
316	lupus anticoagulant (ratio >=1.15)	5.30	43.10	8.13 (0.61 - 108.76)
317	FXIIIA SNP Tyr204phe (dominant)	0.82	9.10	11.1 (5.64 - 21.82)
318	FV Leiden (dominant)	2.40	0.00	0
319	FII SNP G20210A (dominant)	4.00	1.60	0.4 (0 - 77.58)
320	GPIIb SNP ile/Ser (recessive)	1.85	1.20	0.65 (0.18 - 2.37)
321	GPIIIa SNP Leu/pro (recessive)	1.14	1.01	0.89 (0.24 - 3.27)
322	GPIa SNP glu/Lys (recessive)	1.06	0.96	0.91 (0.18 - 4.58)
323	PAI-1 SNP 4G/5G (4G/4G vs 5G/5G)	0.40	0.49	1.23 (0.24 - 6.14)
324	FXIIIA SNP Pro564Leu (dominant)	0.80	0.99	1.24 (0.33 - 4.63)
325	FXIIIA SNP Val34Leu (Val/Leu vs Val/Val)	0.80	1.19	1.49 (0.4 - 5.5)
326	PAI-1 SNP 4G/5G (allele)	0.50	0.84	1.68 (0.45 - 6.24)
327	GPIa SNP C807T (recessive)	1.26	2.24	1.78 (0.45 - 7.04)
328	FXIIIA SNP Tir204Phe (dominant)	1.02	1.95	1.91 (0.2 - 18.29)
329	PAI-1 SNP 4G/5G (4G/5G vs 4G/4G)	0.52	1.10	2.12 (0.51 - 8.69)
330	GPIb SNP thr/Met (recessive)	0.58	1.48	2.55 (0.48 - 13.69)
331	FXIIIA SNP Val34Leu (Leu/Leu vs Val/Val)	0.77	3.59	4.66 (0.44 - 49.1)
332	VWF (Q4 vs Q1)	1.39	1.25	0.9 (0.41 - 1.97)
333	d-dimer (high vs low)	2.10	2.60	1.24 (0.27 - 5.63)
334	FV Leiden (dominant)	0.77	1.12	1.45 (0.16 - 13.07)
335	aPTT (for Protein C) (Q1 vs Q5)	1.53	2.43	1.59 (0.35 - 7.26)
336	VWF SNP P475S (dominant)	0.56	0.98	1.75 (0.1 - 31.18)
337	FXII SNP (dominant)	4.80	4.10	0.85 (0.06 - 12.73)
338	VWF (SD)	1.04	0.97	0.93 (0.65 - 1.34)
339	fibrinogen (SD)	1.13	1.14	1.01 (0.68 - 1.51)
340	d-dimer (SD)	1.02	1.27	1.25 (0.72 - 2.16)
341	t-PA (SD)	1.04	1.60	1.54 (0.55 - 4.34)
342	fibrinogen (Q5 vs Q1)	2.45	1.63	0.67 (0.14 - 3.25)
343	VWF (Q5 vs Q1)	1.52	1.06	0.7 (0.16 - 3.06)
344	d-dimer (Q5 vs Q1)	1.49	1.09	0.73 (0.12 - 4.38)
345	trombin generation (PEAK) (SD)	1.71	1.31	0.77 (0.38 - 1.53)
346	trombin generation (PEAK) (SD)	1.04	1.31	1.26 (0.76 - 2.1)
347	trombin generation (PEAK) (SD)	1.03	1.31	1.27 (0.83 - 1.95)
348	fibrinogen (SD (log scale))	1.52	1.36	0.89 (0.65 - 1.24)
349	FVII:c (unit log) (SD (log scale))	0.98	1.07	1.09 (0.74 - 1.6)
350	t-PA (Q4 vs Q1)	3.20	1.42	0.44 (0.17 - 1.15)
351	d-dimer (Q4 vs Q1)	1.70	1.52	0.89 (0.39 - 2.06)

ID, identification number; RR IS, relative risk for ischaemic stroke; RR MI, relative risk for myocardial infarction; RRR relative risk ratio.
